# Translating spatial transcriptomic signatures in adenosquamous carcinoma into bulk prognostic biomarkers in lung adenocarcinoma: a bottom-up approach

**DOI:** 10.1038/s41698-026-01297-1

**Published:** 2026-01-23

**Authors:** Keiichi Hatakeyama, Takuya Kawata, Koji Muramatsu, Takuma Oishi, Takeshi Nagashima, Ai Sakai, Terumi Nishimura, Keiichi Ohshima, Sumiko Ohnami, Shumpei Ohnami, Yuji Shimoda, Akane Naruoka, Koji Maruyama, Kenichi Urakami, Yasuto Akiyama, Yasuhisa Ohde, Takashi Sugino, Ken Yamaguchi

**Affiliations:** 1 Cancer Multiomics Division, Shizuoka Cancer Center Research Institute, Shizuoka, Japan; 2 Division of Pathology, Shizuoka Cancer Center Hospital, Sunto-gun, Shizuoka, Japan; 3 Cancer Diagnostics Research Division, Shizuoka Cancer Center Research Institute, Shizuoka, Japan; 4 SRL Inc., Shinjuku-ku, Tokyo Japan; 5 Medical Genetics Division, Shizuoka Cancer Center Research Institute, Shizuoka, Japan; 6 Drug Discovery and Development Division, Shizuoka Cancer Center Research Institute, Shizuoka, Japan; 7 Experimental Animal Facility, Shizuoka Cancer Center Research Institute, Shizuoka, Japan; 8 Immunotherapy Division, Shizuoka Cancer Center Research Institute, Shizuoka, Japan; 9 Division of Thoracic Surgery, Shizuoka Cancer Center Hospital, Shizuoka, Japan; 10 Shizuoka Cancer Center, Shizuoka, Japan

**Keywords:** Biomarkers, Cancer, Computational biology and bioinformatics, Oncology

## Abstract

Spatial transcriptomics enables the detection of rare or transitional tumor states not captured by bulk transcriptomics or immunohistochemistry (IHC). However, translating these spatially defined states into clinically relevant biomarkers is challenging, because signals from minor populations are underrepresented or masked in bulk data. Lung adenosquamous carcinoma (ASC), containing intermixed adenocarcinoma and squamous components, provides a model to study lineage transitions and poorly differentiated states unresolved in bulk datasets. Spatial transcriptomic profiling of ASC was integrated with TTF-1 and p40 IHC to detect tumor populations lacking these markers. Gene signatures from IHC-negative populations were projected onto bulk lung adenocarcinoma datasets to assess prognostic relevance. We implemented a color-space uniform manifold approximation and projection visualization (RGB-UMAP) to enhance the spatial mapping of rare transcriptional states that were unresolved by conventional clustering. We identified a TTF-1/p40-negative tumor cell population with a hybrid adenocarcinoma–squamous expression pattern and upregulation of *SLC2A1* (*GLUT1*). RGB-UMAP clarified these states and distinguished them from normal epithelial cells. In bulk lung adenocarcinoma cohorts, high *SLC2A1* expression stratified patients with poorer survival. This study demonstrates a bottom-up biomarker discovery strategy translating spatially defined, IHC-negative tumor cell populations in ASC into stratifiers for lung adenocarcinoma.

## Introduction

Imaging-based spatial transcriptomics (ST) platforms using multiplex RNA fluorescence in situ hybridization (RNA-FISH) enable high-resolution mapping of gene expression at the single-cell level while preserving both tissue architecture and cellular morphology^1,2^. Such platforms can reveal rare and spatially restricted cellular states that remain undetected by bulk transcriptomics or conventional immunohistochemistry (IHC) by maintaining morphological context^3–5^. These elusive cell populations may play key roles in tumor biology, particularly in malignancies with complex histology.

However, despite these advances, imaging-based ST is predominantly used for exploratory characterization, and its direct translation into clinically applicable bottom-up biomarkers is limited. Current approaches for identifying clinically relevant populations often rely on histology-based marker classification or unsupervised clustering based on bulk expression, which can miss transitional or poorly differentiated cell states, particularly in those lacking well-established IHC markers. While computational deconvolution methods can help project single-cell- or high-resolution spot-defined signatures onto bulk transcriptomic datasets, their accuracy heavily relies on the availability of robust reference profiles and tumor content of bulk samples^6^. In this bottom-up process from single-cell spatial profiles to bulk-level patient stratification, nascent screening methods for markers that are minimally affected by tumor content, together with insufficient training datasets that appropriately incorporate conventional histopathological findings, pose a key challenge.

To address one of these challenges, we selected lung adenosquamous carcinoma (ASC) as a model. ASC contains spatially intermixed adenocarcinoma and squamous cell carcinoma components within a single tumor mass, enabling direct comparison of transcriptional and morphological features from distinct lineages within the same tumor microenvironment. Lung ASC, defined as a non-small cell carcinoma comprising both adenocarcinoma and squamous components, is a rare subtype with poor prognosis^7^. Although its pathogenesis remains unclear, cases of histologic transformation of lung adenocarcinoma into squamous cell carcinoma in acquired resistance to EGFR-TKI therapy have been reported^8^. Recent spatial transcriptomics analyses have suggested that ASCs may serve as a model for studying lineage mixing and differentiation states, potentially elucidating the transition from adenocarcinoma to squamous cell carcinoma^9^. Therefore, ASC may serve as a model for investigating the lineage transition of adenocarcinoma toward more malignant squamous phenotypes.

In routine pathological diagnosis of non-small cell lung carcinoma, IHC is commonly performed to distinguish adenocarcinoma from squamous cell carcinoma^10^. TTF-1, encoded by the *NKX2-1* gene, is the most reliable marker of adenocarcinoma, whereas the p40 antibody recognizes the ΔNp63 isoform of p63, highly specific for squamous and basal cells^11,12^. This dual-lineage histology provides an opportunity to systematically identify transitional or poorly differentiated tumor cell states that are difficult to capture in bulk transcriptomic analyses of adenocarcinoma or squamous cell carcinoma alone.

This study aimed to establish a bottom-up biomarker discovery approach that connects single-cell–level spatial profiles to bulk transcriptomes. Specifically, in cases of ASC, we integrated imaging-based spatial transcriptomics with conventional histopathological findings (TTF-1 and p40 immunostaining) and applied a newly developed spatial mapping strategy to exclude normal epithelial cell populations stringently. We extracted candidate genes absent in normal cells but exhibiting spatially stable expression across tumor regions, thereby addressing the challenge of identifying prognostic markers minimally affected by tumor content. Finally, we projected these spatially validated gene signatures onto bulk lung adenocarcinoma datasets to evaluate their association with clinical outcomes. Our approach demonstrates a practical and generalizable strategy for translating spatially resolved single-cell information to clinically relevant bulk-level biomarkers.

## Results

### Overview of the bottom-up biomarker discovery strategy

**Fig. 1:**
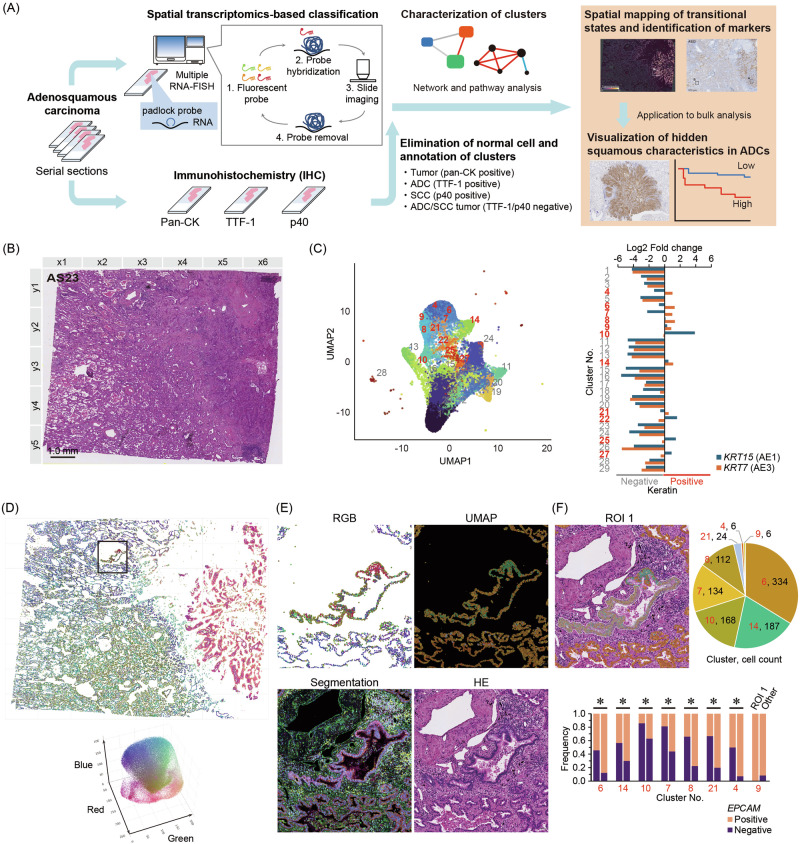
Bottom-up biomarker discovery and spatial clustering-based elimination of normal cell fractions from tumor tissues. **A** Overview of the bottom-up biomarker discovery workflow. Serial formalin-fixed, paraffin-embedded (FFPE) sections of lung adenosquamous carcinoma were analyzed by padlock probe–based spatial transcriptomics (Xenium) and multiplex immunohistochemistry (pan-CK, TTF-1, p40). Pan-CK identified tumor cells, TTF-1 adenocarcinoma (ADC), and p40 squamous cell carcinoma (SCC). After single-cell clustering, normal fractions were excluded, and tumor-enriched clusters were annotated by IHC. Cluster characterization included network and pathway analyses. Spatial mapping revealed transitional ADC/SCC states and candidate markers, subsequently used in bulk datasets to stratify prognosis and visualize hidden squamous features in ADCs. **B** Image of the H&E-stained section of lung ASC (AS23). **C** UMAP-based dimensionality reduction and clustering using Xenium data. Cluster numbers are ordered by descending cell count. Keratin-positive clusters are shown in red. Reference cell counts for each cluster are provided in Supplementary Fig. S2A. **D** RGB-UMAP visualization of keratin-positive clusters. Three-dimensional UMAP coordinates were scaled to the RGB color space and mapped back to tissue coordinates. Black box marks the region shown in (**E**). **E** Multimodal imaging of the boxed region, including RGB-UMAP, standard UMAP, Xenium segmentation with fluorescence imaging, and the corresponding H&E staining. In the segmentation panel, DAPI staining is shown in blue, ATP1A1/CD45/CDH1 signals are depicted in magenta, αSMA/VIM in green, and 18S rRNA in yellow. **F** Cluster composition and EPCAM-positive cell ratio in region of interest (ROI) 1 compared with other regions. ROI 1 is marked with a white, manually drawn boundary. Asterisk indicates statistical significance (*p < 0.05).

We developed a framework that integrated ST profiling with histological annotation to initiate bottom-up biomarker discovery. As illustrated in Fig. 1A, we first performed unsupervised spatial clustering of single-cell transcriptomes obtained from lung ASC tissues. To map clusters onto the tissue structure, an hematoxylin and eosin (H&E)-stained section from the identical sample was prepared and used as a spatial reference (Fig. 1B). Serial sections were subjected to IHC for pan-keratin, TTF1, and p40, to identify tumor regions and distinguish adenocarcinoma from squamous components (Fig. S1). Clusters with normal-like features were excluded based on marker gene expression and histological context. The remaining tumor-enriched clusters were annotated using IHC-defined histological subtypes. We extracted squamous-feature-associated genes from these annotated clusters and validated them in an independent ASC sample. Finally, we evaluated the clinical significance of these spatial transcriptomic markers in bulk RNA microarray data from patients with lung adenocarcinoma, which was supported by survival and pathway analyses. These sequential steps correspond to the overall workflow summarized in Fig. 1A.

### Identification of spatially distinct keratin-positive tumor clusters

We performed unsupervised clustering of single-cell transcriptomic profiles obtained via the Xenium platform to characterize the cellular heterogeneity within tumor tissues. uniform manifold approximation and projection (UMAP)-based dimensionality reduction revealed 27 transcriptionally distinct clusters (Fig. 1C), numbered in descending order of total cell count. We evaluated keratin gene expression across clusters to identify epithelial tumor populations. Clusters exhibiting a positive log_2_ fold change in keratin expression were classified as keratin-positive and are highlighted in red. Notably, these keratin-positive clusters localized together on the UMAP plot, suggesting a shared transcriptomic phenotype.

Furthermore, we evaluated the cytological and transcriptional characteristics of keratin-positive clusters to support their identity as tumor-enriched epithelial populations. Keratin-positive clusters exhibited a significantly higher proportion of multinucleated cells compared with keratin-negative clusters (Fig. S2A), along with increased nuclear and cellular areas (Fig. S2B). These clusters demonstrated a significantly greater total transcript count per cell (Fig. S2C), indicative of elevated transcriptional activity. EPCAM expression was enriched in regions densely populated by keratin-positive clusters on the UMAP plot, supporting their epithelial and likely neoplastic nature (Fig. S2D). Assuming tumor content corresponds to the proportion of keratin-positive cells, the estimated tumor fraction from spatial transcriptomics was consistent with the tumor purity derived from whole-exome sequencing (WES) (Fig. S2E). These cytological and molecular features support the interpretation that keratin-positive clusters represent tumor-enriched epithelial cell populations within ASC.

These findings suggest that transcript counts increase with expanding cell area. As the Xenium platform enables spatially resolved quantification of gene expression per cell area, we sought to minimize the confounding influence of cell size when comparing samples or genes. Therefore, transcript counts were normalized by cell area and expressed as counts per cell per 100 μm^2^. The distribution of normalized expression values across clusters is shown in Fig. S3.

### RGB-UMAP mapping highlights rare cell populations

We applied RGB-UMAP mapping based on single-cell gene expression profiles to visualize spatial heterogeneity within keratin-positive clusters (Fig. 1D). Gene expression data were first reduced to three dimensions using UMAP, and each of the resulting components was mapped to a red, green, or blue color channel. This encoding produced composite colors for individual cells, such that transcriptionally similar cells appeared in similar colors. This visualization enabled intuitive identification of rare or distinct cell states not evident from clustering alone.

Our H&E-stained sample (AS23) contained two regions identified as normal lung epithelium by clinical pathology. One representative area is highlighted with black frames in Fig. 1D, and enlarged views are shown as multimodal images generated from spatial transcriptomic and protein expression data in Fig. 1E. In standard UMAP embedding, cells from these normal epithelial regions were included within the same cluster as surrounding tumor cells, making them indistinguishable by clustering alone. In contrast, RGB-UMAP mapping revealed distinct coloration in these cells, reflecting transcriptomic divergence from nearby tumor cells. Based on this distinction, we defined this region as Region of Interest 1 (ROI 1) and quantified both the cluster composition and EPCAM positivity rate within this ROI (Fig. 1F). ROI 1 was enriched in keratin-positive clusters otherwise identified as tumor-associated in other tissue regions. However, *EPCAM* positivity was significantly lower in ROI 1 compared to other keratin-positive regions, except for cluster 9. Similarly, the other area with normal epithelial cells was evaluated as ROI 2 and showed the same trend (Fig. S4). This suggests that clustering based on transcriptomic features and on epithelial tumor markers, such as EPCAM, alone may be insufficient to exclude normal epithelial cells completely.

Notably, RGB-UMAP enabled the identification of these normal cells through color-guided spatial mapping, independent of pathologist input. These findings highlight the utility of RGB-UMAP as a powerful visualization tool for integrating spatial transcriptomic and cytological information to refine tumor annotation and exclude contaminating populations. In subsequent analyses, cells in ROIs 1 and 2, identified as histologically normal epithelial cells, were excluded to ensure tumor-specific interpretation of spatial transcriptomic data. Accordingly, keratin-positive cells were considered tumor cells, whereas keratin-negative cells were classified as non-tumor cells henceforth.

### Spatial visualization for integration with IHC

**Fig. 2:**
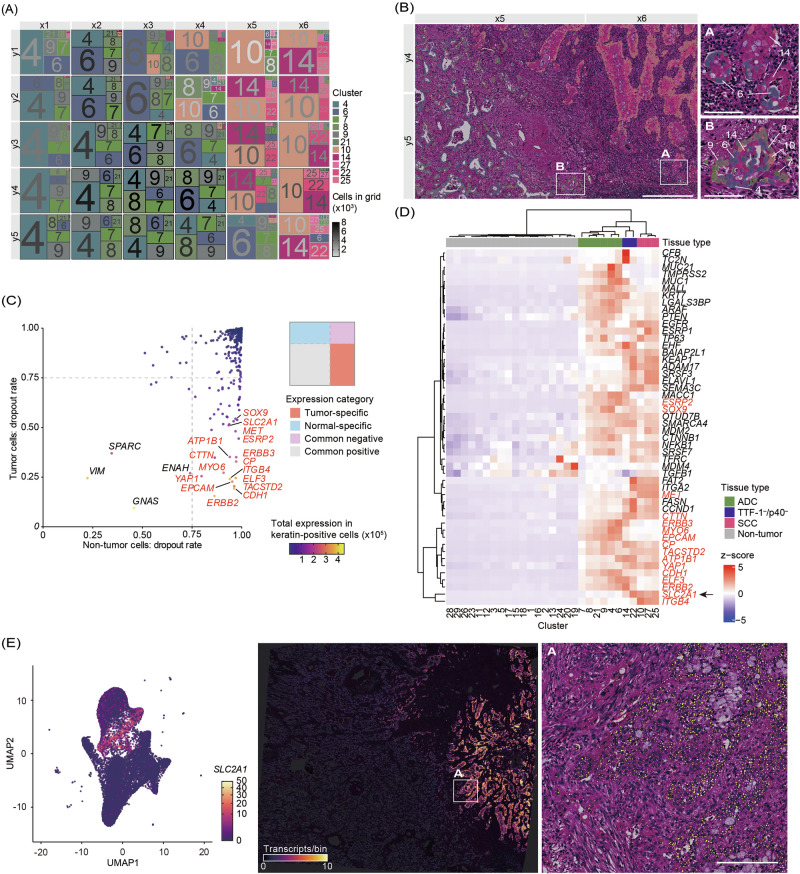
Spatial visualization of keratin-positive clusters and classification of gene expression patterns. **A** A treemap showing the spatial distribution of tumor-enriched clusters. Each block corresponds to a spatial region defined in Fig. 1B. Block colors represent the mean RGB value of cells within each cluster, based on the RGB-UMAP in Fig. 1D. The font color of the cluster numbers indicates the number of tumor-enriched cells in the respective block. **B** Clusters overlaid on representative H&E-stained tissue sections. White boxes indicate the areas enlarged in (**A**, **B**). Cluster numbers are shown only in the magnified images and are colored to match the treemap in (**A**). Scale bars represent 500 µm (overview) and 100 µm (insets). **C** Gene classification based on dropout rates in tumor (keratin-positive) and non-tumor (keratin-negative) cells. Each dot represents a gene, plotted as the dropout rate in non-tumor (x-axis) against tumor-enriched (y-axis) cells. Genes are grouped into tumor-specific (red), normal-specific (blue), common negative (gray), and common positive (purple) categories based on 75% dropout thresholds (dotted lines). The top 20 tumor-specific genes are labeled, with red indicating the tumor-specific category. **D** Heatmap of tumor-specific genes across clusters. Clusters are annotated by tissue type based on immunohistochemistry. Non-tumor clusters are designated as non-tumor. Genes labeled in red correspond to those in (**C**). An arrow is used to highlight *SLC2A1* within the heatmap. **E** UMAP and spatial distribution of *SLC2A1* expression. Expression is shown by UMAP embedding (left) and on spatial tissue coordinates (right). The white-outlined region is enlarged to show *SLC2A1* expression signals as yellow dots overlaid on the H&E-stained section. Scale bar = 200 µm.

We visualized the distribution of tumor-enriched (keratin-positive) clusters using a treemap format to improve the spatial interpretation of UMAP-based clustering (Fig. 2A). Each block in the treemap corresponds to a spatial region defined in Fig. 1B and is colored according to the mean RGB-UMAP color of the cells within each cluster. The font color of each cluster number represents the relative abundance of cells in that block. This spatial representation revealed distinct localization patterns across the tissues of tumor-enriched clusters. Clusters 10, 14, 22, 25, and 27 were predominantly localized in areas with p40 staining, consistent with squamous histology, whereas clusters 4, 6, 7, 8, 9, and 21 were in regions of TTF-1 positivity, consistent with adenocarcinoma components (see Fig. S1). Regions containing clusters with contrasting RGB-UMAP color tones were frequently observed near the histological interface between adenocarcinoma and squamous cell carcinoma components (Fig. 2B). Based on IHC profiles, clusters 14 and 22 were negative for both TTF-1 and p40, which may indicate poorly differentiated or transcriptionally ambiguous epithelial cell states. Although TTF-1 negativity was due to the presence of intracellular mucin, morphologically, these clusters were considered adenocarcinoma.

### Identification of markers specific to squamous cell carcinoma components

To identify genes specifically expressed in tumor-enriched cells, we first classified all detected genes into four categories based on their dropout rates in tumor versus non-tumor cells (Fig. 2C). The dropout rate was defined as the proportion of cells in which gene expression was undetectable. Among genes showing high expression in tumor cells, some, including *VIM*, *SPARC*, *GNAS*, and *ENAH*, were frequently expressed in non-tumor cells and were thus categorized as common-positive. Their broad expression patterns were confirmed by widespread distribution across clusters in the UMAP embedding (Fig. S5). In contrast, among the tumor-specific genes defined by dropout rate, those with high total expression in keratin-positive tumor cells (top 20 genes labeled in Fig. 2C, of which 16 classified as tumor-specific are highlighted in red) were selected for downstream analyses. We constructed a heatmap using this refined tumor-specific gene set, excluding the four common-positive genes, to visualize their distribution across clusters (Fig. 2D). Clusters were annotated based on histological subtypes defined by IHC. Consistent with their tumor-enriched detection pattern, these tumor-specific genes showed markedly lower average expression in most non-tumor clusters, whereas higher z-scores were observed in tumor-enriched clusters. A few genes (e.g., *TFRC*, *MDM4*, *TGFB1*) exhibited locally elevated z-scores in small non-tumor clusters (clusters 20, 24, and 19; Supplementary Fig. S2); however, these clusters contain very few cells, and the apparent elevation reflects the gene-wise z-score representation rather than substantial expression within non-tumor populations. This also highlights that dropout-based classification alone cannot fully exclude rare gene signals in small non-tumor clusters, further supporting the need for histology-guided interpretation in subsequent analyses. Based on hierarchical clustering in the heatmap, the TTF-1⁻/p40⁻ cluster exhibited a transcriptional profile more similar to adenocarcinoma (ADC)-associated clusters than to squamous cell carcinoma (SCC) clusters. We evaluated the expression of these genes in histologically normal epithelial cells, excluded as ROIs 1 and 2. While several genes highly expressed in tumor-enriched clusters were detected in normal lung epithelium, *SLC2A1* was barely expressed, suggesting its specificity for tumor-enriched populations (Fig. S6). *SLC2A1* expression was represented in the UMAP embedding, suggesting its largely restricted expression to tumor-enriched clusters (Fig. 2E). *SLC2A1*-positive cells were localized in the SCC component on spatial tissue maps. Overlay of H&E-stained sections and expression patterns confirmed restricted *SLC2A1* expression to histologically defined tumor areas. These findings indicate that *SLC2A1* is predominantly associated with SCC components, with additional expression in TTF-1⁻/p40⁻ clusters.

### Validation of tumor-specific gene signatures

**Fig. 3:**
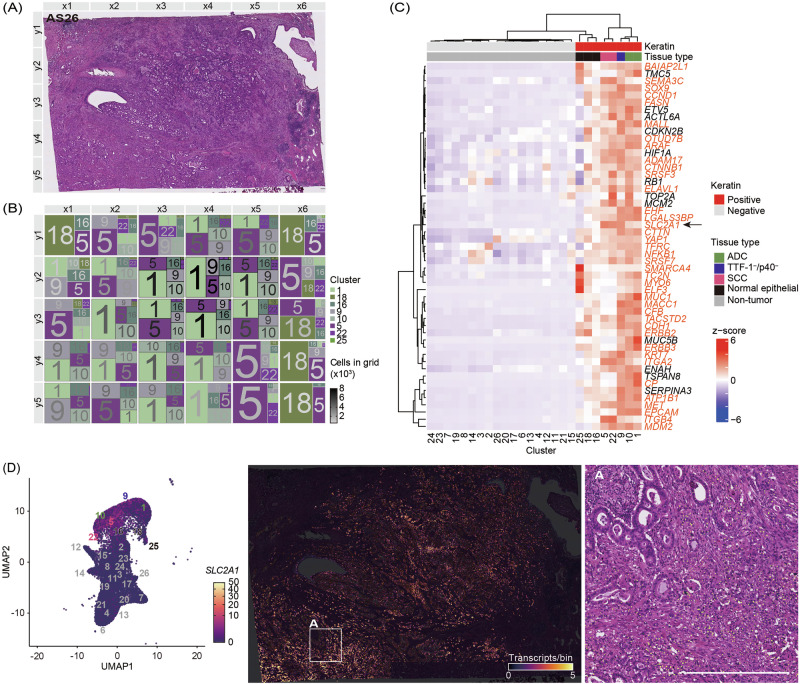
Validation of tumor-specific gene signatures in an independent lung adenosquamous carcinoma sample. **A** Image of a H&E-stained section of lung adenosquamous carcinoma for validation (AS26). **B** A treemap showing the spatial distribution of keratin-positive clusters. Each block corresponds to a spatial region defined in (**A**). Block colors represent the mean RGB value of cells within each cluster based on the RGB-UMAP in Fig. S7. The font color of the cluster numbers indicates the number of keratin-positive cells in the respective block. Because the RGB-UMAP embedding is computed independently for each sample, similarities in colors across samples do not directly correspond to similarities in gene-expression profiles; the colors reflect transcriptomic relationships within each sample. **C** Heatmap of tumor-specific genes across clusters. Clusters are annotated by tissue type based on immunohistochemistry. Keratin-negative clusters are designated as non-tumor. Among the keratin-positive clusters, those histologically identified as normal epithelial cell are annotated as normal. Tumor-specific genes common in Fig. 2D are labeled in orange. An arrow is used to highlight *SLC2A1* within the heatmap. **D** UMAP and spatial distribution of *SLC2A1* expression. Expression is shown on a UMAP embedding (left) and on spatial tissue coordinates (right). The white-outlined region is enlarged to show *SLC2A1* expression signals as yellow dots overlaid on the H&E-stained section. Scale bar = 500 µm.

We analyzed an independent case of lung ASC to validate the robustness of tumor-specific gene signatures identified in the primary sample (Fig. 3A). As in the discovery dataset, serial sections were subjected to IHC staining for TTF-1, p40, and pan-keratin to delineate adenocarcinoma, squamous, and tumor regions, respectively (Fig. S7). Spatial transcriptomic data were processed by UMAP-based clustering, followed by the extraction of keratin-positive clusters. These clusters were visualized using RGB-UMAP (Fig. S7). In this sample, ADC and SCC components were colored in green and purple, respectively. This color-coded spatial heterogeneity is reflected in the treemap (Fig. 3B), revealing that most observation areas contain intermixed ADC and SCC components.

Dropout analysis was performed to identify genes specific to tumor subtypes, and their expression patterns were visualized using a heatmap (Fig. 3C). Clusters 16, 18, and 25 were morphologically identified as keratin-positive normal lung epithelial cells, and tumor subtypes were annotated based on IHC. In the discovery sample (AS23), keratin-positive normal lung epithelial cells were present only in small, scattered foci and did not form distinct clusters, whereas in the validation sample (AS26) it occupied a larger continuous area and was therefore resolved as separate keratin-positive normal clusters. Consistent with the discovery sample (AS23), the TTF1⁻/p40⁻ cluster in the validation case exhibited a transcriptomic profile closer to ADC than SCC. Among the tumor-specific genes, 39 genes (highlighted in blue), including *SLC2A1*, were shared between the primary and validation samples. The complete list of 39 tumor-specific genes shared between AS23 and AS26, along with subgroup-wise positive cell counts, is provided in Supplementary Table S3. *SLC2A1* expression was lower in keratin-positive normal epithelial clusters compared to tumor-enriched clusters. In Fig. 3D, *SLC2A1* expression is projected onto the UMAP embedding and spatial tissue map. *SLC2A1*-positive cells were enriched in regions with a high density of tumor-enriched (keratin-positive) clusters and predominantly localized to the SCC component than the ADC region. These findings reinforce the tumor-restricted expression pattern of *SLC2A1* across independent ASC samples.

To further evaluate inter-patient reproducibility of SLC2A1 expression patterns, we analyzed five additional ASC cases (AS32, AS36, AS37, AS38, and AS41) using serial immunohistochemistry for pan-keratin, TTF-1, p40, and SLC2A1 together with spatial mapping of *SLC2A1* mRNA expression (Supplementary Fig. S8). In all cases, SLC2A1 protein staining and spatial mRNA expression localized to p40-positive squamous regions as well as TTF-1⁻/p40⁻ tumor areas, showing highly consistent spatial distribution across patients. These findings confirm that SLC2A1 expression in these tumor compartments is reproducible across independent ASC cases and extend the robustness of our observations beyond the initial discovery and validation samples analyzed by spatial transcriptomics.

### Differential expression of tumor-specific genes in histological subtypes

**Fig. 4:**
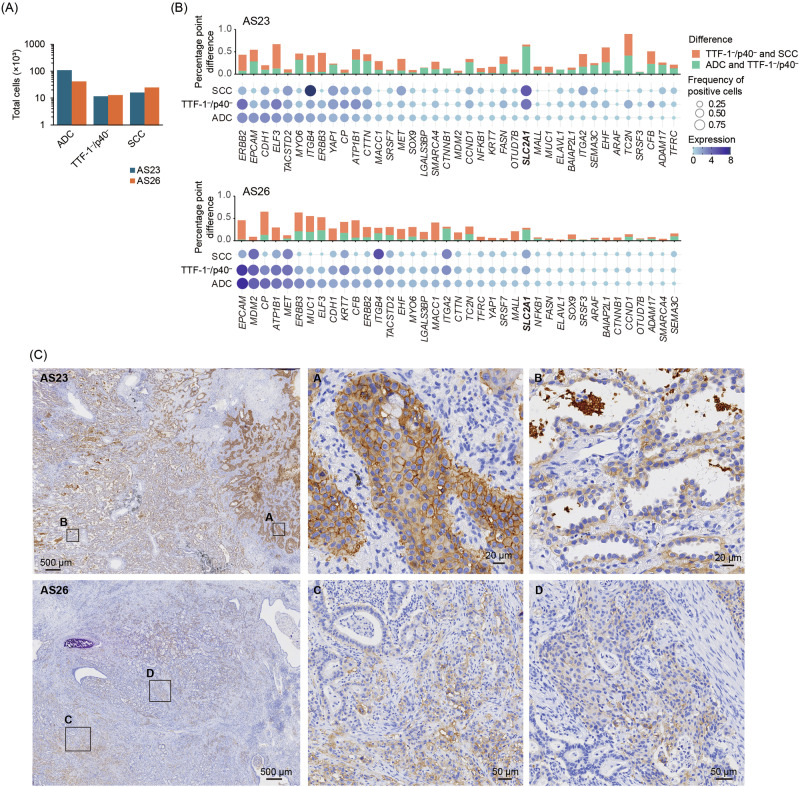
Expression patterns of tumor-specific genes across histological subtypes in two adenosquamous cell carcinoma samples. **A** Total number of cells classified into three subtypes (ADC, TTF-1⁻/p40⁻, and SCC) in AS23 and AS26 samples. **B** Dot plots displaying the frequency (dot size) and average expression (color intensity) of each gene in ADC, TTF-1⁻/p40⁻, and SCC subtypes. Genes are sorted in descending order of frequency in the ADC subtype. The selected SLC2A1 markers validated by immunohistochemistry (IHC) are indicated in bold. Above the dot plots, bar graphs indicate the difference in the percentage of gene-expression–positive cells between subtypes. Green bars represent the difference between ADC and TTF-1⁻/p40⁻, and orange bars represent the difference between TTF-1⁻/p40⁻ and SCC. **C** Validation of SLC2A1 expression by IHC. Representative IHC-stained sections from AS23 and AS26 are shown. **A**, **C** show membranous or cytoplasmic staining in tumor epithelial regions. **B**, **D** show negative or weakly positive regions, consistent with spatial heterogeneity. The insets in the leftmost, low-magnification views indicate regions magnified in the right-hand panels.

To dissect the expression patterns of tumor-specific genes between histological subtypes, we compared transcriptomic profiles of three distinct tumor categories (ADC, TTF-1⁻/p40⁻, and SCC) in both the discovery (AS23) and validation (AS26) samples. First, we quantified the number of cells classified into each subtype (Fig. 4A). In both samples, ADC constituted the majority of tumor cells, while the proportion of SCC and TTF-1⁻/p40⁻ cells was comparatively higher in AS26 than in AS23. Next, we visualized the frequency and expression of 39 tumor-specific genes that were commonly detected in both samples using bubble plots, alongside bar graphs representing differences in positive cell proportions between subtypes (Fig. 4B). Genes highly expressed in ADC cells were also expressed in TTF-1⁻/p40⁻ cells, suggesting that these populations may share certain transcriptional programs. For example, *ERBB2/3*, typically downregulated in SCC, showed larger differences between SCC and TTF-1⁻/p40⁻ than between ADC and TTF-1⁻/p40⁻, supporting a relatively closer expression pattern between the latter two. In contrast, *SLC2A1* expression increased from ADC to TTF-1⁻/p40⁻.

To validate SLC2A1 expression at the protein level, we performed IHC for serial sections from AS23 and AS26 (Fig. 4C). Strong membranous or cytoplasmic staining was observed in morphologically squamous regions, whereas adenocarcinoma regions and normal epithelial cells showed minimal or no expression. Thus, SLC2A1 expression is largely confined to the squamous component, including TTF-1⁻/p40⁻ cells, and the TTF-1⁻/p40⁻ population may represent a unique group with transcriptional features that partially overlap with both ADC and SCC.

### From spatially single-cell-level insights to bulk validation

Squamous features are often partially intermixed within the tumor, even in tumors histologically diagnosed as adenocarcinoma. Based on our spatial transcriptomic findings, we hypothesized that a subset of TTF-1-negative and p40-negative cells exhibiting characteristics of both ADC and squamous cell carcinoma may exist in conventional ADC. Therefore, we analyzed the relationship between bulk *SLC2A1* expression and molecular profiles of ADC cases.

**Fig. 5:**
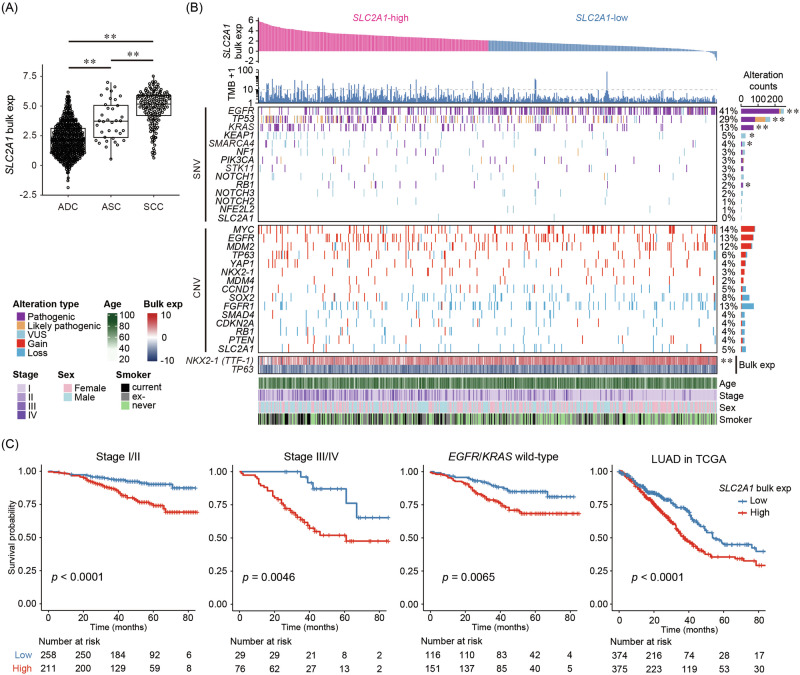
*SLC2A1* bulk expression and molecular profiling in lung adenocarcinoma (ADC). **A** *SLC2A1* bulk expression across histological subtypes. ASC, adenosquamous carcinoma; SCC, squamous cell carcinoma. Asterisk indicates statistical significance (**p < 0.01). **B** Genomic landscape and clinical features stratified by *SLC2A1* expression. Samples are sorted by bulk *SLC2A1* expression (from high to low), with groups divided at the median. Panels include tumor mutation burden (TMB), single-nucleotide variants (SNVs), copy number variations (CNVs), bulk expression of *NKX2-1* (TTF-1) and *TP63*, and clinical annotations (age, stage, sex, and smoking history). Genes with significantly different frequencies between ADC and SCC were selected. The rightmost panel shows the frequency and cumulative count of SNVs and CNVs, with asterisks indicating genes with significant differences between *SLC2A1*-high and -low groups (*p < 0.05, **p < 0.01, Fisher’s exact test). Exp expression, VUS variant of uncertain significance. **C** Kaplan–Meier survival analysis stratified by *SLC2A1* bulk expression in ADC patients. Overall survival curves showing the comparison of the *SLC2A1*-high (red) and *SLC2A1*-low (blue) groups in our cohort and the TCGA lung adenocarcinoma (LUAD) cohort. Our cohort was analyzed in the following subsets defined by clinical characteristics: stage I/II, stage III/IV, and *EGFR*/*KRAS* wild-type. Statistical significance was assessed using the log-rank test. The number of patients at risk is shown below each plot.

As shown in Fig. 5A, *SLC2A1* expression was significantly higher in SCC and ASC compared to ADC (*p* < 0.01), consistent with spatial transcriptomic and IHC findings in our discovery and validation samples. ADC samples were further stratified into high and low SLC2A1 expression groups using the median value as a cutoff (Fig. 5B). In the high *SLC2A1* expression group, tumor mutation burden (TMB) increased significantly, along with a higher frequency of somatic mutations in *TP53*, *KEAP1*, and *RB1*, known to be frequently altered in squamous cell carcinoma. In contrast, the low expression group showed a significant enrichment of *EGFR* mutations and higher expression of *NKX2-1* (*TTF-1*), consistent with features typically observed in Asian ADC cases. A weak inverse relationship between *SLC2A1* and *NKX2-1* was observed (Fig. S9A, *p* < 0.01). While these trends suggest a possible association between elevated *SLC2A1* expression and squamous-like molecular features, *KRAS* mutations, rare in squamous cell carcinoma, were also enriched in the high expression group. Thus, *SLC2A1*-high ADCs may comprise a heterogeneous group including tumors with varying degrees of squamous and adenocarcinoma molecular characteristics. In line with these molecular features, glycolysis-related genes were significantly upregulated in the *SLC2A1*-high group compared with the *SLC2A1*-low group (Fig. S9B).

Finally, we assessed the prognostic significance of *SLC2A1* expression in ADC. Kaplan–Meier survival analyses demonstrated that patients with high *SLC2A1* expression exhibited significantly poorer overall survival in both TCGA lung adenocarcinoma dataset and our cohort (Fig. 5C). Clinical characteristics of patients included in the overall survival cohort are summarized in Table S2, comprising 574 ADC and 148 SCC patients. This association remained significant in clinically relevant subgroups, including patients in stage III/IV and those wild-type for *EGFR* and *KRAS*. In contrast, in SCC, no significant prognostic difference was observed (Fig. S9C). To assess whether *SLC2A1* remained associated with survival after adjusting for clinical factors, we performed a multivariable Cox proportional hazards analysis including pathological stage, age, smoking status, and gender (Fig. S10). This association remained significant in the multivariable model, confirming *SLC2A1* as an independent prognostic factor (HR 2.64, 95% CI 1.68–4.14, *p* < 0.001).

To investigate whether bulk *SLC2A1* expression reflected its protein-level expression in tumor tissues, we performed IHC in independent ADC cases stratified by bulk *SLC2A1* levels (Fig. S11). In the high-expression case (AD364), strong cytoplasmic and membranous staining was observed, consistent with the transcriptomic data. In contrast, the low-expression case (AD139) showed weak focal staining. These results provide additional histological support for the correlation between SLC2A1 expression and tumor phenotypes at the protein level.

### Network and pathway analysis for SLC2A1-related genes

**Fig. 6:**
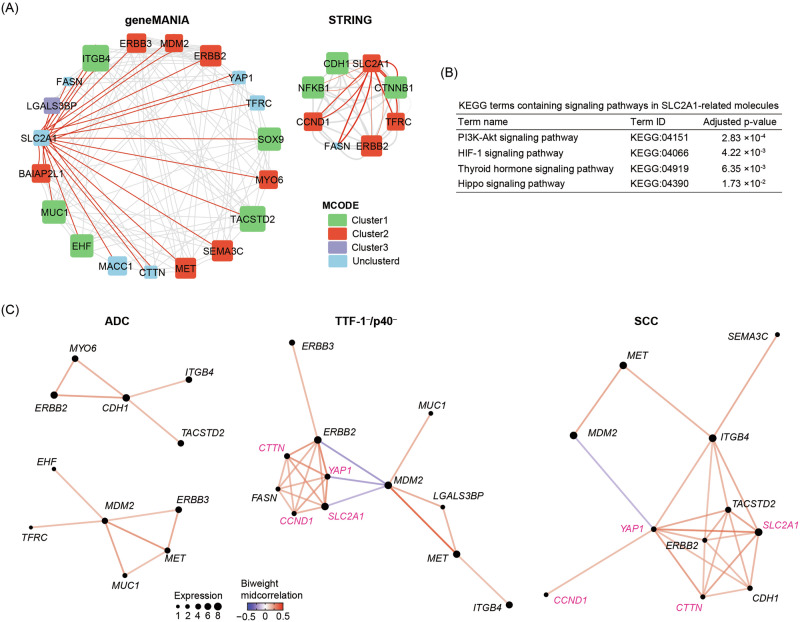
Network and pathway analysis for SLC2A1-related genes. **A** Gene association network from GeneMANIA (left) and protein–protein interaction network from STRING (right) for genes related to SLC2A1. Of the 39 tumor-specific genes, 23 were connected in these networks. Edges indicate known or predicted interactions; edges directly linked to SLC2A1 are red. Molecules were clustered using MCODE in Cytoscape, and colored accordingly: Cluster 1 (green), Cluster 2 (blue), Cluster 3 (red), Unclustered (light blue). **B** KEGG signaling pathways enriched among SLC2A1-related genes, identified by GeneMANIA and STRING, and analyzed using g:Profiler. This pathway analysis is based on the 23 SLC2A1-related genes connected in the GeneMANIA/STRING networks shown in (**A**). **C** Gene coexpression networks of SLC2A1-related genes based on biweight midcorrelation (bicor) in ADC, TTF-1⁻/p40⁻, and SCC subtypes. These coexpression networks are based on the 23 SLC2A1-related genes identified in (**A**). Networks were constructed based on single-cell gene expression data. Node size represents gene expression, and edge color indicates the strength and direction of bicor. Magenta-colored genes indicate those commonly coexpressed in subgroups exhibiting squamous epithelial features (TTF-1⁻/p40⁻ and SCC).

We conducted network and pathway analyses to elucidate the molecular context of SLC2A1-related genes in different tumor subtypes. First, we constructed a gene coexpression network (GeneMANIA) and a PPI network (STRING) of 39 tumor-specific genes commonly expressed across samples, and extracted 23 genes directly connected to SLC2A1 in either network (Fig. 6A). These molecules spanned multiple MCODE-defined clusters, indicating involvement in distinct molecular modules. Next, we performed pathway analysis using g:Profiler for the 23 SLC2A1-related molecules extracted from both networks and identified significantly enriched KEGG signaling pathways (Fig. 6B). In parallel, Gene Ontology Biological Process (GO:BP) enrichment highlighted epithelial-associated biological programs—including epithelial differentiation, epithelium morphogenesis, cell adhesion, and tissue remodeling as driver terms (Supplementary Table S4). These GO terms collectively reflect both epithelial maintenance pathways typically active in adenocarcinoma and cytoskeletal/adhesion remodeling pathways enriched in squamous carcinoma, consistent with the mixed transcriptional phenotype observed in the TTF-1⁻/p40⁻ population. Finally, to examine subtype-specific coexpression patterns, we computed biweight midcorrelation (bicor) values for these 23 genes in ADC, TTF-1⁻/p40⁻, and SCC cell populations based on single-cell gene expression data (Fig. 6C). Distinct coexpression networks were observed across subtypes. In SCC and TTF-1⁻/p40⁻, *SLC2A1* showed strong coexpression with *YAP1*, *CCND1*, and *CTTN*, a pattern not observed in ADC. These genes are involved in the pathways identified in the enrichment analysis (Fig. 6B), suggesting that the observed coexpression patterns may reflect activation of these pathways in SCC and TTF-1⁻/p40⁻ subpopulations.

## Discussion

We established a bottom-up biomarker discovery strategy, where spatially defined single-cell tumor states were translated into clinically relevant bulk-level markers. In this study, keratin-positive cells were designated as the primary tumor fraction for spatial transcriptomic analysis. This designation showed consistent morphological, molecular, and spatial findings. Keratin-positive clusters exhibited cytological features characteristic of neoplastic epithelial populations, including a higher prevalence of multinucleated cells, increased nuclear and cellular areas, and elevated total transcript counts per cell. Multinucleation is expected to reflect aberrant mitotic events or cell–cell fusion during tumor growth^13^. Similar associations between enlarged cell size and increased transcriptional output have been reported in several tumors, including transcriptional amplification driven by oncogenic factors, such as MYC, which increases the per-cell abundance of expressed mRNAs^14–16^. Recent spatial transcriptomics studies suggest that tumor cells often display larger dimensions and higher transcript counts than surrounding non-neoplastic cells^17^. Results of keratin-positive clusters showed enriched *EPCAM* expression, and the estimated tumor fraction based on these cells closely matched tumor purity values from WES. Although some keratin-positive clusters were present in histologically normal lung epithelium, RGB-UMAP visualization enabled their identification based on transcriptomic divergence from tumor cells, allowing their exclusion from downstream analyses. These results provide a robust, literature-supported basis for defining keratin-positive cells as the tumor fraction, ensuring that subsequent analyses reflect tumor-specific biology with minimal contamination from non-neoplastic epithelial populations.

In our Xenium datasets, keratin-positive tumor cells exhibited larger cellular areas and higher total transcript counts per cell compared with keratin-negative cells. Transcript counts in part of our analysis were normalized by cell area and expressed as counts per 100 μm^2^ to minimize size-related confounding effects, thereby emphasizing expression density rather than gross transcript abundance. This adjustment, consistent with current recommendations for imaging-based spatial transcriptomics^18^, was applied to the RGB-UMAP visualization, reducing the impact of tumor cell hypertrophy on expression patterns and, consequently, improving the accuracy of subtype comparisons.

In our study, RGB-UMAP revealed histologically normal epithelial cells embedded within keratin-positive clusters that would otherwise be classified as tumors based solely on conventional clustering and keratin expression, despite being clearly recognizable as normal epithelial cell on pathology. These cells exhibited distinct coloration patterns corresponding to transcriptomic differences from surrounding tumor cells, enabling their precise delineation without prior pathological annotation. RGB-UMAP is conceptually reminiscent of the Vesalius approach, where spot-level transcriptomic data are mapped to the RGB color space to create visually segmented tissue territories without relying on clustering algorithms^19^. However, unlike Vesalius, RGB-UMAP retains single-cell resolution and directly overlays these color annotations onto the original spatial coordinates. This allowed intuitive detection of rare cell populations or histologically ambiguous cells that diverge from their surroundings, as illustrated in Figs. 1E and S 4B. Furthermore, incorporating expression data normalized by cell area reduces the influence of cell size and minimizes artifacts from morphological variation. Collectively, these features establish RGB-UMAP as a robust complementary tool to conventional clustering methods, enhancing the resolution and accuracy of tumor–normal discrimination in spatial transcriptomic analyses.

SLC2A1 is more highly expressed in lung squamous cell carcinoma than in lung adenocarcinoma^18^, and high expression in the latter is associated with poor prognosis and resistance to taxanes^20,21^. In this study, we identified high *SLC2A1* expression in the TTF-1⁻/p40⁻ subgroup, exhibiting both adenocarcinoma- and squamous-type transcriptional features, a mixed phenotype further supported by pathway analyses. High expression was observed in p40-positive squamous components, supporting the view that *SLC2A1* overexpression is not confined to the TTF-1⁻/p40⁻ subgroup but is a broader feature of squamous-like phenotypes. These observations were supported by network analysis (Fig. 6C), suggesting strong *SLC2A1* co-expression with *YAP1*, *CCND1*, and *CTTN* in TTF-1⁻/p40⁻ and SCC subgroups. These genes are potentially important hubs in the PI3K–Akt, HIF-1, and Hippo signaling pathways, all implicated in tumor progression and metabolic adaptation^22–24^.

In lung ASC, tumor regions are not always clearly demarcated into adenocarcinoma and squamous components; instead, they can also present as areas of indistinct admixture. In our AS23 and AS26 cases, p40-positive squamous components coexisted with TTF1/p40-negative regions, a distribution previously described as a mucoepidermoid carcinoma (MEC)-like pattern^25^, in which spatial transcriptomics demonstrated that the TTF1/p40-negative component exhibits morphological and molecular characteristics resembling those of adenocarcinoma. This finding provides supportive evidence for the long-standing hypothesis that the squamous component of lung ASC may arise through squamous transformation from adenocarcinoma. Given the admixture of adenocarcinoma and squamous elements in ASC, high SLC2A1 expression in adenocarcinoma likely reflects the presence of a minor SCC-like subpopulation analogous to the TTF-1⁻/p40⁻ component.

SLC2A1 (GLUT1) is a well-established HIF-1 target and integral to hypoxia-driven glycolytic remodeling^26,27^. Consistent with this knowledge, our pathway analysis using the tumor-specific gene set associated with SLC2A1 revealed enriched HIF-1 signaling, which is closely linked to hypoxic responses (Fig. 6B). Hypoxia facilitates adenocarcinoma-to-squamous transformation in pancreatic cancer models^28^. Therefore, hypoxic conditions may favor squamous transformation within ASC, thereby contributing to the observed upregulation of SLC2A1.

Frost et al. reported the association of loss of TTF-1 (*NKX2-1*) expression with poor prognosis and reduced efficacy of pemetrexed-based chemotherapy. They suggested that TTF-1–negative adenocarcinomas may represent a subtype that mimics the biological features and behavior of squamous cell carcinoma^29^. In our study, *SLC2A1*, expressed in both the TTF-1/p40-negative and SCC regions of ASC, was highly expressed in a subset of ADC, and this subpopulation showed reduced TTF-1 expression. These findings strongly suggest the presence of adenocarcinoma cells with squamous-like properties concealed within the ADC. The causal relationship between TTF-1 loss and SLC2A1 upregulation is unclear and further functional studies are required to establish mechanistic links.

Consistent with these observations, glycolysis-related genes were significantly upregulated in *SLC2A1*-high ADCs (Fig. S9B), suggesting a shift toward a more glycolytic metabolic state, consistent with the hypoxia-induced upregulation of SLC2A1 mediated by HIF-1^26,30^. This metabolic pattern parallels the glycolytic dependency characteristic of lung squamous cell carcinoma, in which SLC2A1-driven glucose uptake supports tumor bioenergetics and survival^31^. These findings raise the possibility that a subset of *SLC2A1*-high ADCs may share similar metabolic vulnerabilities, including potential sensitivity to SLC2A1/GLUT1 inhibition or glycolytic pathway targeting.

These findings suggest the presence of a subpopulation within lung adenocarcinoma characterized by high *SLC2A1* expression and acquisition of squamous-like traits, which may define a more aggressive tumor phenotype and contribute to the poorer prognosis observed in bulk analyses. Our bottom-up biomarker discovery approach provides a means to identify biomarkers that capture heterogeneous cell populations while simultaneously offering insights into the underlying molecular mechanisms.

While our bottom-up biomarker discovery approach enabled the identification of heterogeneous cell populations and provided molecular insights, some limitations warrant further consideration. First, the analysis was based on a limited number of spatial transcriptomics (Xenium) cases and external validation is needed to confirm whether the identified *SLC2A1*-high, squamous-like subpopulation is broadly observed across patients. Second, the approach is inherently constrained by the targeted gene panel used for spatial profiling, limiting the analysis to a pre-selected set of transcripts, excluding novel markers, splicing variants, mutated transcripts, and non-coding RNAs. Third, the biological roles of candidate genes and pathways were inferred from co-expression and network analyses, and functional studies should establish causality. Finally, clinical translation requires quantitative assessment of how such spatially defined subpopulations contribute to bulk tumor signatures, including signals from normal cells, and prognosis, as well as validation in larger, multi-institutional cohorts.

We established a bottom-up biomarker discovery approach that translates rare, spatially defined transcriptional states identified in ASC using spatial transcriptomics and IHC into prognostically relevant signatures for lung adenocarcinoma. We demonstrated that gene programs derived from histologically and transcriptionally distinct subpopulations can retain clinical significance even within homogenized datasets by integrating high-resolution spatial mapping with bulk transcriptomic projection. This strategy offers a practical pathway to connect spatial and bulk molecular data, capturing intratumoral heterogeneity for precision oncology and supporting the development of clinically relevant stratification markers in lung cancer.

## Methods

### Ethics statement

All procedures involving human participants were conducted in accordance with the ethical standards of the institutional and national research committees and with the principles of Declaration of Helsinki and its later amendments. The Institutional Review Board of the Shizuoka Cancer Center (approval number: 25–33) approved the study. All analyzed samples were retrospectively obtained from formalin-fixed, paraffin-embedded (FFPE) tissues. Written informed consent was obtained from all patients, including consent for the possibility of secondary findings such as those arising from blood-based constitutional analyses. The ages of the patients who underwent spatial analysis in the discovery and validation cohorts were 79 and 85 years, respectively. Clinical trial registration was not applicable.

### Sample preparation and spatial transcriptomics

Two surgically resected cases of lung ASC were analyzed. FFPE tissue blocks were serially sectioned at 5 μm thickness. Total RNA was extracted from deparaffinized FFPE tissue sections using the RNeasy FFPE kit for RNA extraction (Qiagen, Hilden, Germany), and the DV200 value was measured using a TapeStation system (Agilent Technologies, CA, USA) to check its quality. Both cases showed DV200 values greater than 30. One section per case was used for spatial transcriptomic analysis on the Xenium v1 platform (10x Genomics, Pleasanton, CA, USA) with Xenium Cell Segmentation Staining Reagents, and performed in a single run on the same slide, while serial sections were subjected to H&E staining and IHC for thyroid transcription factor-1 (TTF-1), p40, and pan-keratin. The Xenium assay and imaging were performed following the manufacturer’s protocol, targeting approximately 400 genes from the Xenium Human Lung Cancer Expression Panel supplemented with custom add-on probe panels (Table S1).

### Image registration and histological annotation

H&E- and IHC-stained slides were scanned at high resolution (NanoZoomer, Hamamatsu Photonics, Shizuoka, Japan) and converted to the OME-TIFF format using QuPath (version 0.5.0). These images were co-registered with the corresponding Xenium spatial transcriptomic images using Xenium Explorer (version 3.2.0)^32^. Tumor regions, including normal epithelial cells, were annotated histologically based on combined marker expression and morphological features by a pulmonary pathologist as ADC, SCC, or TTF-1-negative/p40-negative (TTF-1⁻/p40⁻) subtype. Because Xenium profiling was performed on a single 5-µm FFPE section, adjacent serial H&E and IHC slides were used for histological annotation. Although individual cells cannot be matched one-to-one across serial sections, the use of adjacent IHC sections for lineage determination is standard diagnostic pathology practice. Tumor classification is made at the regional level, and regional correspondence provides robust annotation for defining ADC, SCC, and TTF-1⁻/p40⁻ areas in the Xenium dataset.

### Processing of spatial transcriptomic data

Transcript count matrices and cell-wise metadata (including spatial coordinates, cell and nuclear areas, and total transcript counts) were obtained from the Xenium output files and imported into R (version 4.4.1) using Seurat (version 5.0) and custom scripts. Clustering was performed using the standard Xenium pipeline, which applies the Leiden community detection algorithm to principal component analysis (PCA)-reduced gene expression data.

Keratin-positive and keratin-negative cells were identified from transcriptomic profiles by calculating fold changes in keratin gene expression (*KRT15*/*KRT7*) after UMAP followed by Leiden clustering (as implemented in the Xenium on-board pipeline). To maintain consistency with routine pathological practice, in which pan‑keratin (AE1/AE3) staining delineates epithelial tumor regions, we used the epithelial keratins KRT7 and KRT15 available in the Xenium panel. KRT7 is a low‑molecular‑weight keratin commonly used as a marker of adenocarcinoma, while KRT15 is associated with squamous epithelial lineages; together they approximate the keratin classes typically encompassed by AE1/AE3 staining and were therefore adopted as the most appropriate panel‑available epithelial markers to define the keratin‑positive compartment.

### RGB-UMAP spatial mapping

Gene expression was normalized by cell area and scaled to counts per 100 μm^2^ to control for size-dependent effects. For visualization, a two-dimensional embedding was generated using UMAP based on the PCA-reduced space.

We implemented an RGB-UMAP visualization approach to enhance the detection of rare cells with distinct transcriptional signatures. Normal cells without keratin expression were excluded before dimension reduction. Gene expression data were reduced to three UMAP components, each rescaled in the range of 0–255 and mapped to the red, green, and blue color channels, respectively. Each cell was thus assigned a composite RGB color simply representing its transcriptional identity. Color-coded cells were overlaid on the original tissue coordinates to generate spatial maps, enabling visual discrimination of transcriptionally distinct cell populations, including rare subsets not clearly separated by conventional clustering.

### Network and pathway analysis

A gene network was constructed from the identified marker genes using GeneMANIA, with the maximum number of resultant genes set to zero to restrict the network to the input list. A protein–protein interaction (PPI) network was generated from the same gene set using STRING (high confidence, interaction score ≥0.700). Cluster analysis of the resulting networks was performed using the MCODE plugin in Cytoscape. Pathway enrichment analysis was conducted using g:Profiler, and Benjamini–Hochberg adjusted *p*-values < 0.05 were considered statistically significant.

Single-cell gene co-expression networks were constructed separately for ADC, SCC, and TTF-1⁻/p40⁻ cell populations. Normalized expression matrices for each group were extracted and combined from two Xenium datasets. Pairwise gene–gene correlations were calculated using the biweight midcorrelation (bicor) method implemented using the WGCNA R package, which is robust to outliers in single-cell expression data.

### Bulk transcriptomic and genomic data analysis

Bulk RNA microarray expression data and WES data were obtained from our previously published institutional cohort comprising 574 lung cancer cases^33,34^ and are available in the NBDC Human Database repository (https://humandbs.dbcls.jp; accession ID hum0127.v4). TCGA lung adenocarcinoma RNA-seq data and corresponding clinical information (>700 cases) were downloaded from the UCSC Xena platform^35,36^ and were used for survival analysis.

### **IHC analysis**

Immunostaining was performed using an automated stainer (Bond-III, Leica Microsystems, Tokyo, Japan) with heat-induced antigen retrieval in BOND Epitope Retrieval Solution 1 (Leica Microsystems) for 20 min at 100 °C following the manufacturer’s protocol. The following primary antibodies were used: mouse monoclonal anti-human TTF-1 (clone 8G7G3/1, M3575, Agilent Technologies; dilution 1:500), p40 (polyclonal, ACR3030B, Biocare Medical, CA, USA; dilution 1:200), pan-keratin (clone AE1/AE3, IR053, Agilent Technologies; ready-to-use), and SLC2A1 (polyclonal, ab15309, Abcam, Cambridge, UK; dilution 1:400).

### Validation of SLC2A1 expression in additional ASC cases

To assess the inter-patient reproducibility of SLC2A1 expression patterns identified in the discovery (AS23) and validation (AS26) Xenium datasets, five additional surgically resected ASC cases (AS32, AS36, AS37, AS38, and AS41) were analyzed using serial immunohistochemistry and spatial mRNA mapping. FFPE sections were stained for pan-keratin (AE1/AE3), TTF-1, p40, and SLC2A1 following the same staining conditions described above. Corresponding *SLC2A1* mRNA spatial maps were generated for each case using single-gene visualization based on the Xenium Human Lung Cancer Panel. These datasets were used to confirm the spatial distribution of SLC2A1 within tumor compartments, including adenocarcinoma, squamous, and TTF-1⁻/p40⁻ regions.

### Statistical analysis

All statistical analyses were performed using Excel (version 2507), R (version 4.4.1), and Python (version 3.13). For survival analysis, Kaplan–Meier curves were generated and compared using the log-rank test. Between-group comparisons were performed using Welch’s *t*-test and Fisher’s exact test for categorical variables. A two-sided *p* < 0.05 was considered statistically significant. The Benjamini–Hochberg method was used for multiple testing correction. A multivariable Cox proportional hazards model adjusted for clinical covariates (pathological stage, age, smoking status, and gender) was additionally performed for survival analysis.

## Supplementary information

Supplementary information

## Data Availability

WES and microarray data have been deposited in the NBDC as Controlled-Access Data (Research ID: hum0127.v4; https://humandbs.biosciencedbc.jp/en/). Other datasets generated and/or analyzed during the current study, including spatial transcriptomic data, are not publicly available due to ethical and institutional restrictions, but are available from the corresponding author upon reasonable request.

## Code availability

All analysis scripts used in this study are available from the corresponding author upon reasonable request.
