# Supplementary material for: Translating spatial transcriptomic signatures in adenosquamous carcinoma into bulk prognostic biomarkers in lung adenocarcinoma: a bottom-up approach
Source: NPJ Precis Oncol. 2026 Jan 23;10:77. doi: 10.1038/s41698-026-01297-1 (PMC12929729; doi:10.1038/s41698-026-01297-1)
Supplement: Supplementary file 1 — Supplementary information [file 41698_2026_1297_MOESM1_ESM.pdf]

## Supporting information

**Supplementary Figure S1.** Immunohistochemical staining for histological annotation of spatial clusters.
**Supplementary Figure S2.** Cytological and molecular features of keratin-positive clusters support their tumor specificity.
**Supplementary Figure S3.** Distribution of transcript counts per cell area in each cluster.
**Supplementary Figure S4.** RGB-UMAP-based detection of normal epithelial cells in a second region (ROI 2).
**Supplementary Figure S5.** UMAP plots showing the expression of common-positive genes (*VIM, SPARC*, *GNAS*, and *ENAH*) identified by dropout analysis.

**Supplementary Figure S6.** Heatmap of tumor-specific gene expression counts in normal epithelial cells.

**Supplementary Figure S7.** Immunohistochemical staining and RGB-UMAP of validation sample for histological annotation of spatial clusters.

**Supplementary Figure S8.** Inter-patient validation of SLC2A1 expression patterns in TTF-$1^{-}$/p$40^{-}$ tumor regions using immunohistochemistry and spatial mRNA mapping.

**Supplementary Figure S9.** Relationship between *SLC2A1* bulk expression, *NKX2-1* (*TTF-1*) bulk expression, and glycolysis-related gene bulk expression in lung adenocarcinoma (ADC), and survival analysis for squamous cell carcinoma (SCC).

**Supplementary Figure S10.** Multivariable Cox proportional hazards analysis of overall survival in lung adenocarcinoma.

**Supplementary Figure S11.** Immunohistochemical validation of SLC2A1 expression in ADC cases.

**Supplementary Table S1.** Probe information for Xenium spatial transcriptomics.

**Supplementary Table S2.** Characteristics of patients with adenocarcinoma (ADC) and squamous cell carcinoma (SCC) along with their survival outcomes.

**Supplementary Table S3.** Positive cell counts of the 39 tumor-specific genes across adenocarcinoma (ADC), TTF-$1^{-}$/p$40^{-}$, and squamous cell carcinoma (SCC) subgroups in the discovery (AS23) and validation (AS26) samples.

**Supplementary Table S4.** Gene Ontology (GO) Biological Process enrichment analysis in SLC2A1-related molecules.

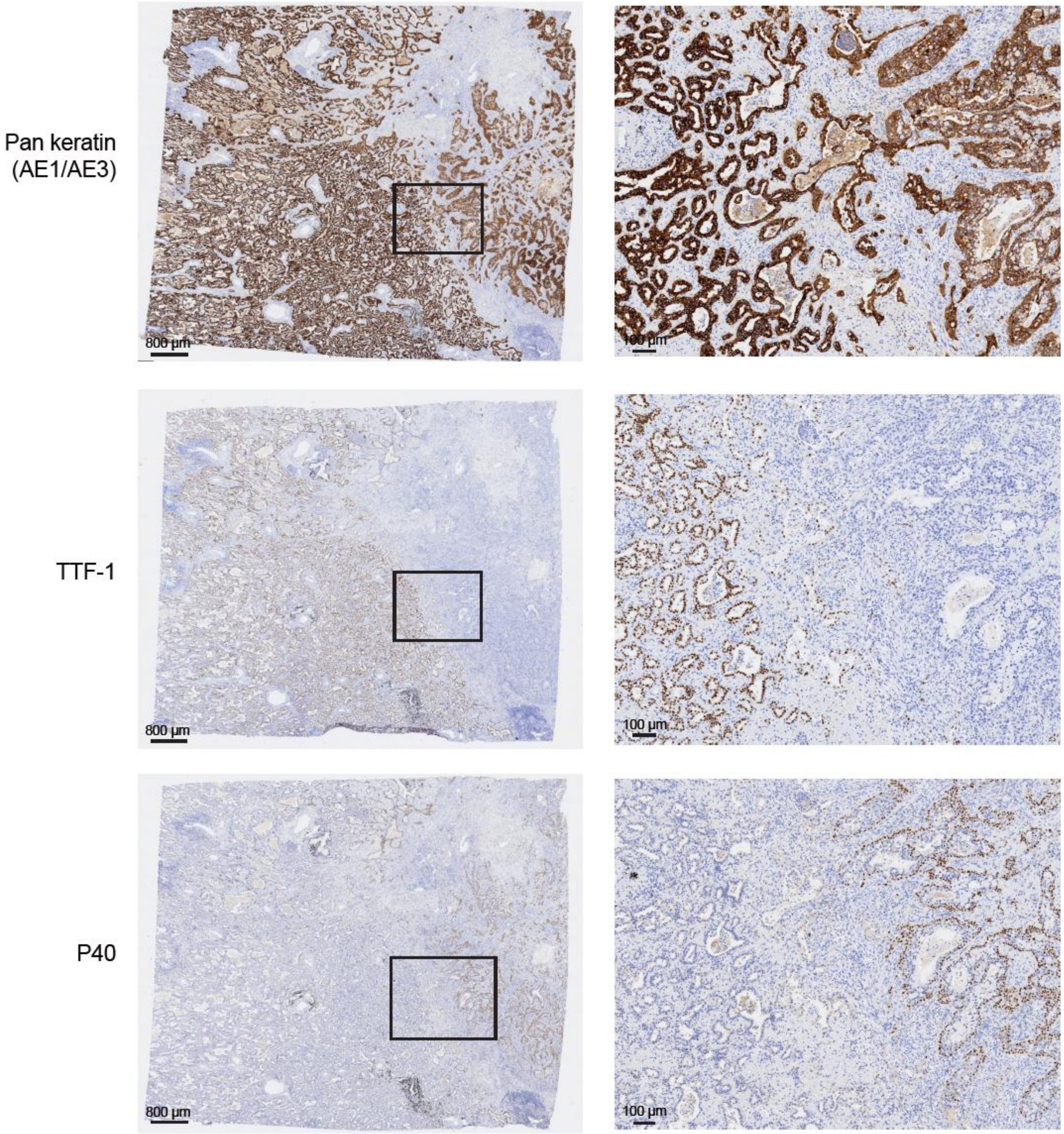


**Supplementary Figure S1. Immunohistochemical staining for histological annotation of spatial clusters.** Serial sections from the adenosquamous carcinoma tissue (sample no, AS23) shown in Figure 1 were stained for TTF-1, p40, and pan-keratin. These markers defined adenocarcinoma (TTF-1-positive), squamous (p40-positive), and tumor (keratin-positive) regions, supporting the histological annotation of spatial clusters identified by transcriptomic profiling.

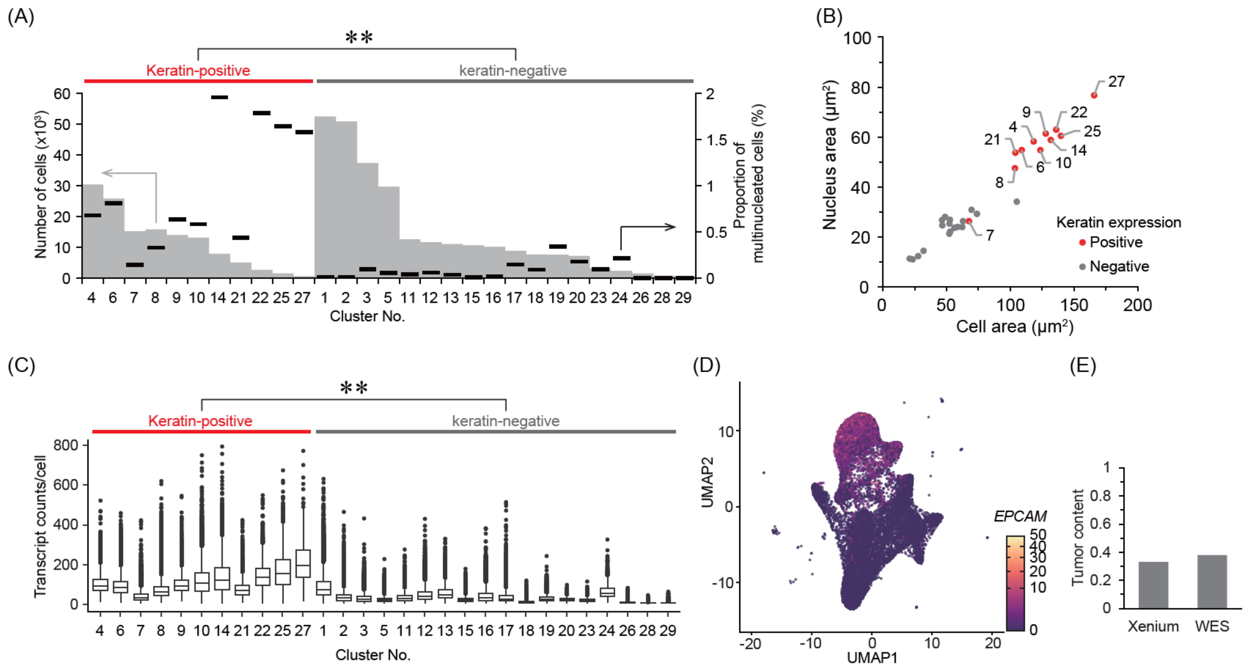


**Supplementary Figure S2. Cytological and molecular features of keratin-positive clusters support their tumor specificity.** (A) Number of cells and proportion of multinucleated cells in each cluster. The total number of analyzable cells in the discovery sample (AS23) was 398,136. Cluster labels follow the original Xenium on-board clustering based on total raw cell counts. In this study, cells that passed the vendor-recommended quality-control filters were defined as analyzable cells; therefore, discrepancies may occur between the original cluster numbering and the number of analyzable cells within each cluster. (B) Comparison of nuclear area and total cell area between keratin-positive and keratin-negative clusters. Cell and nucleus identification were performed using Xenium's standard cell segmentation algorithm. Names of keratin-positive clusters are indicated in the figure. (C) Total transcript count per cell in keratin-positive versus keratin-negative clusters. (D) Distribution of *EPCAM* expression overlaid on the UMAP plot. Color intensity reflects expression counts at the single-cell level. (E) Comparison of tumor fraction estimated from keratin-positive cell proportions and tumor purity calculated from whole-exome sequencing (WES). Keratin-positive cells represented 33.1% of all cells, whereas 66.9% （approximately 266,000 cells） were keratin-negative and considered non-tumor fractions. Asterisks indicate statistical significance (** $p < 0.01$)

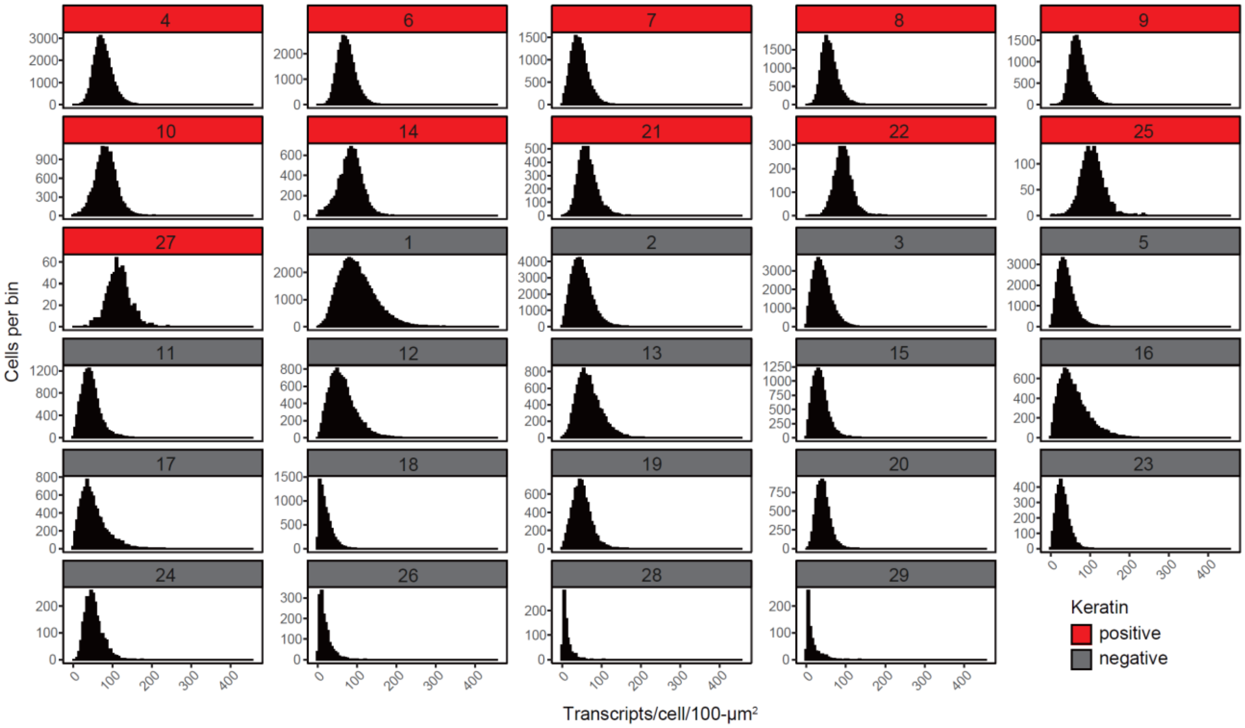


**Supplementary Figure S3. Distribution of transcript counts per cell area in each cluster.** Histograms showing the distribution of normalized transcript counts (transcripts per cell per 100 $\mu m^2$) for each cluster. Normalization was performed by dividing the total transcript counts per cell by cell-area and scaling to 100 $\mu m^2$ to adjust for cell size differences. Clusters are ordered and labeled by cluster number. Red headers indicate keratin-positive clusters, while gray headers denote keratin-negative clusters.

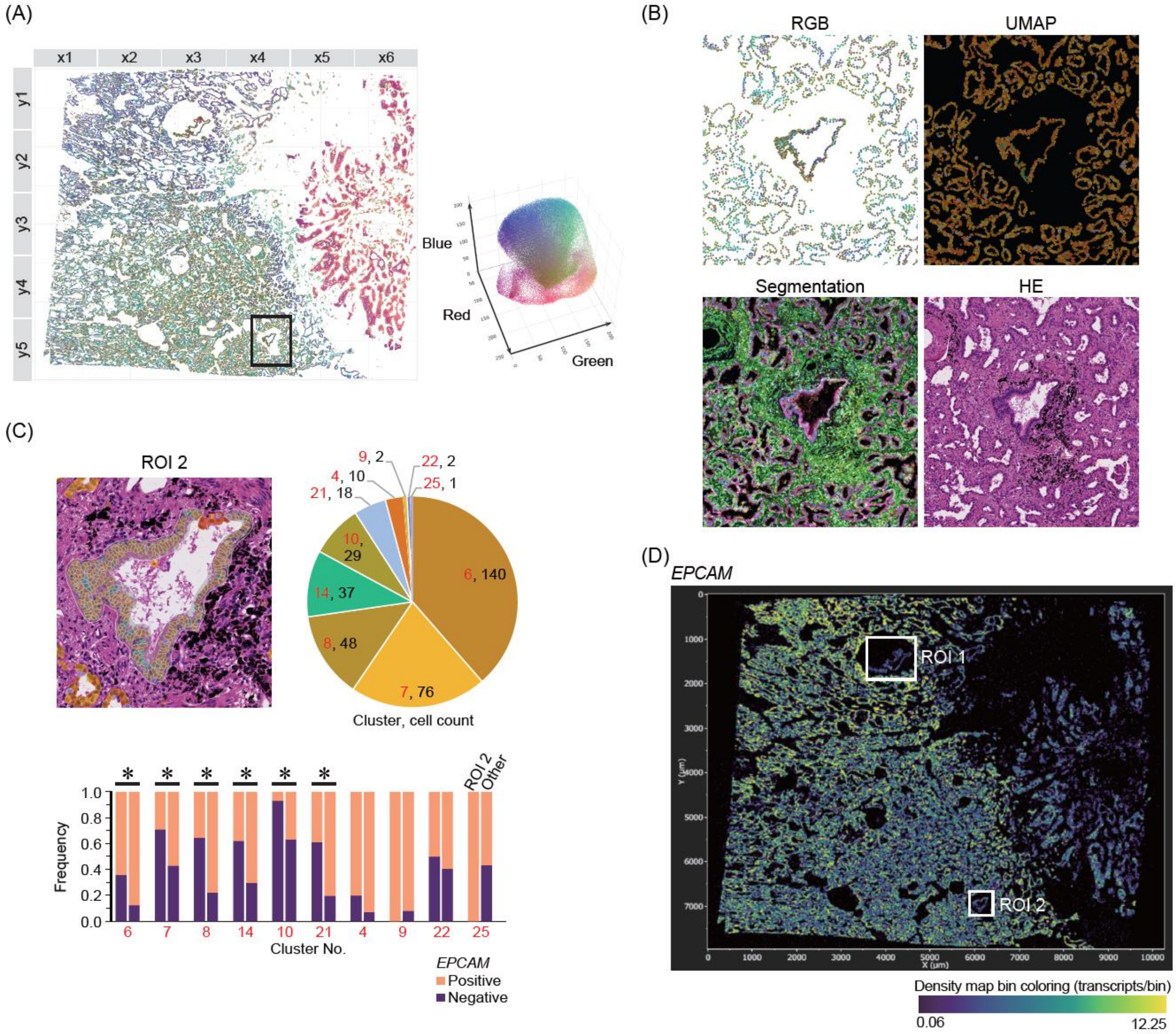

**Supplementary Figure S4. RGB-UMAP-based detection of normal epithelial cells in a second region (ROI 2).** (A) RGB-UMAP visualization of keratin-positive clusters. Three-dimensional UMAP coordinates were scaled to RGB color space and mapped back to tissue coordinates. Black box marks the region shown in (B). (B) Multimodal imaging of the boxed region, including RGB-UMAP, standard UMAP, Xenium segmentation with fluorescence imaging, and the corresponding H&E staining. In the segmentation panel, DAPI staining is shown in blue, ATP1A1/CD45/CDH1 signals are depicted in magenta, αSMA/VIM in green, and 18S rRNA in yellow. (C) Cluster composition and EPCAM-positive cell ratio in region of interest (ROI) 2 compared with other regions. ROI 2 is marked with a white manually drawn boundary. Asterisk indicates statistical significance (*$p < 0.05$). (D) Density map of *EPCAM* expression. Regions containing ROI 1 and ROI 2 are marked by white boxes. *EPCAM* expression is lower in both ROI 1 and ROI 2 compared to the surrounding tumor regions, consistent with their identification as normal epithelial areas.

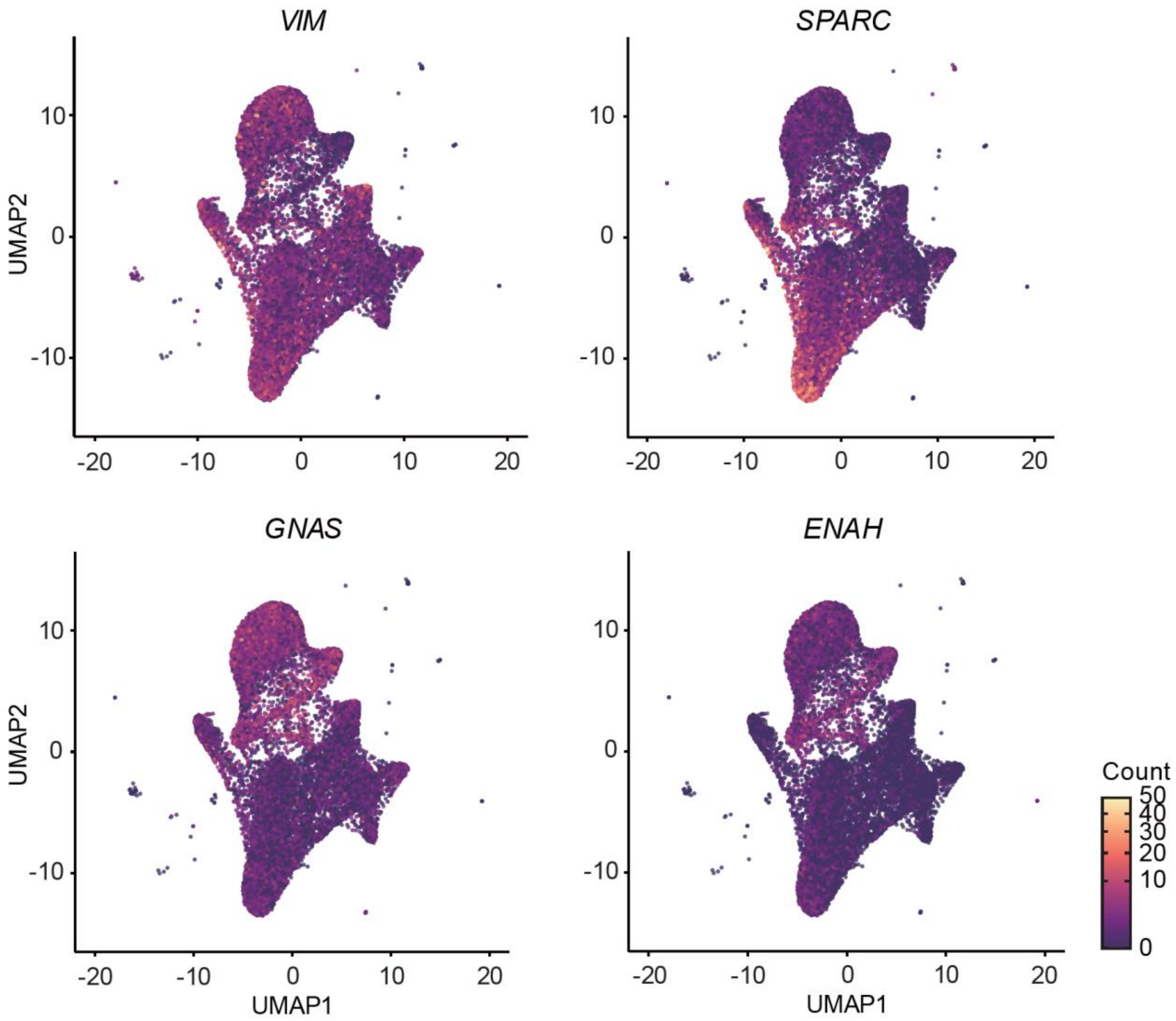


**Supplementary Figure S5. UMAP plots showing the expression of common-positive genes (*VIM, SPARC*, *GNAS*, and *ENAH*) identified by dropout analysis.** Color scale indicates transcript counts per cell.

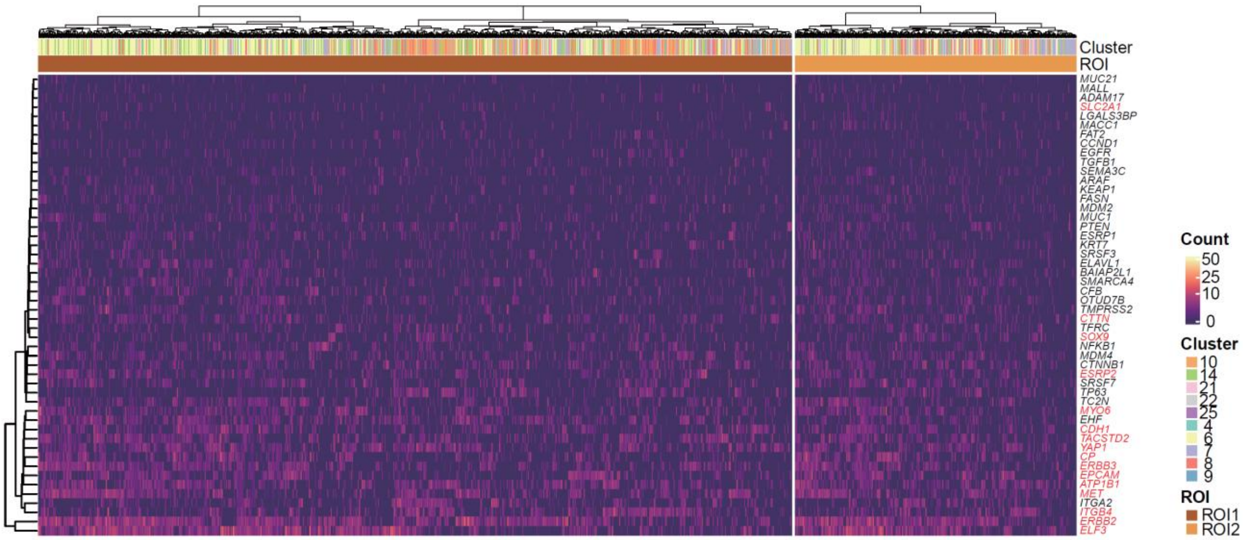


**Supplementary Figure S6. Heatmap of tumor-specific gene expression counts in normal epithelial cells.** Tumor-specific genes were assessed in histologically normal epithelial regions (ROI 1 and ROI 2) which were excluded from tumor-enriched regions during analysis. Color scale indicates transcript counts per cell.

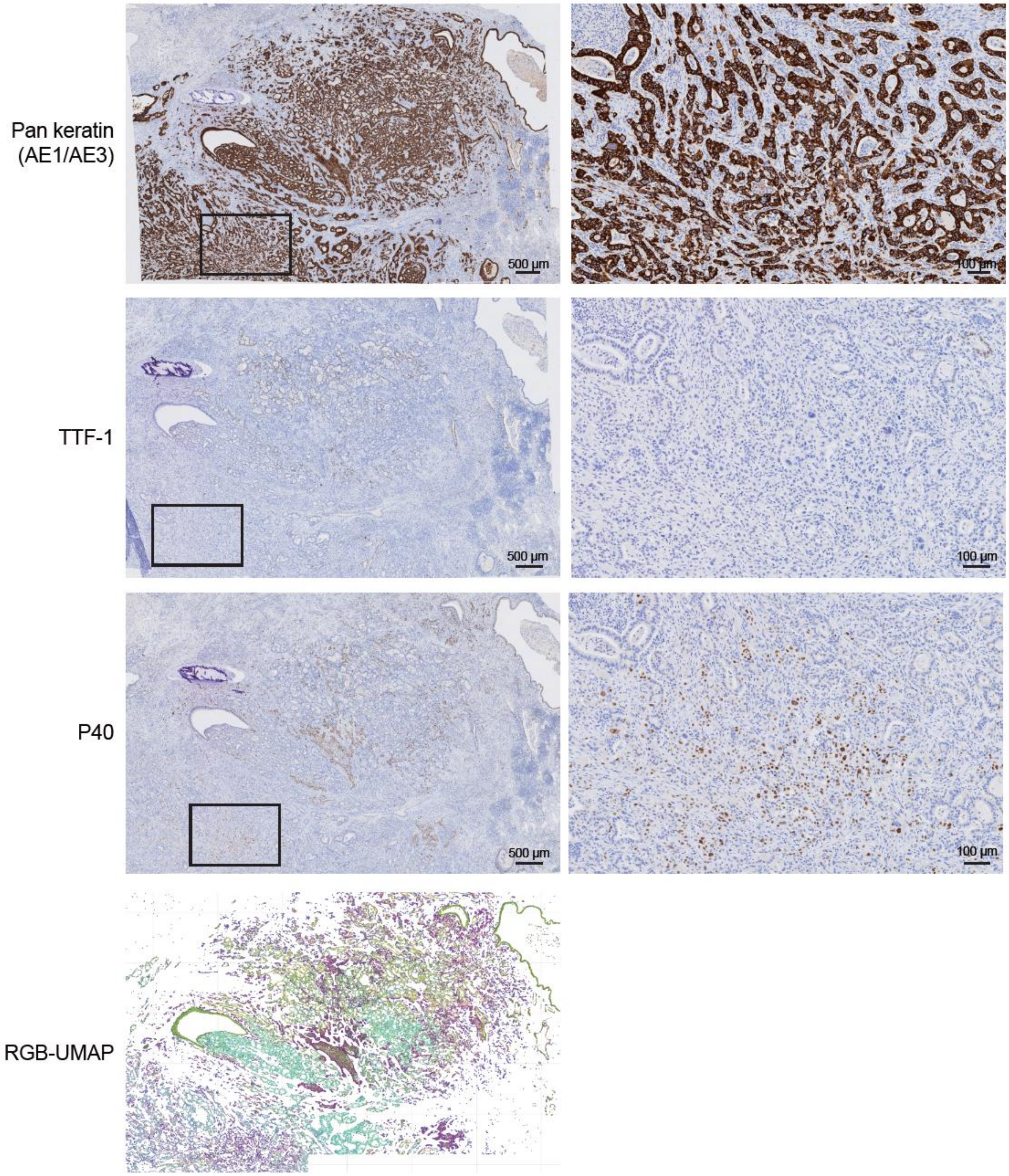


**Supplementary Figure S7. Immunohistochemical staining and RGB-UMAP of validation sample for histological annotation of spatial clusters.** Serial sections from the adenosquamous carcinoma tissue (sample no, AS26) for validation shown in Figure 3 were stained for TTF-1, p40, and pan-keratin. These markers defined adenocarcinoma (TTF-1-positive), squamous (p40-positive), and tumor (keratin-positive) regions, supporting the histological annotation of spatial clusters identified by transcriptomic profiling. RGB-UMAP visualization based on keratin-positive clusters in this sample.

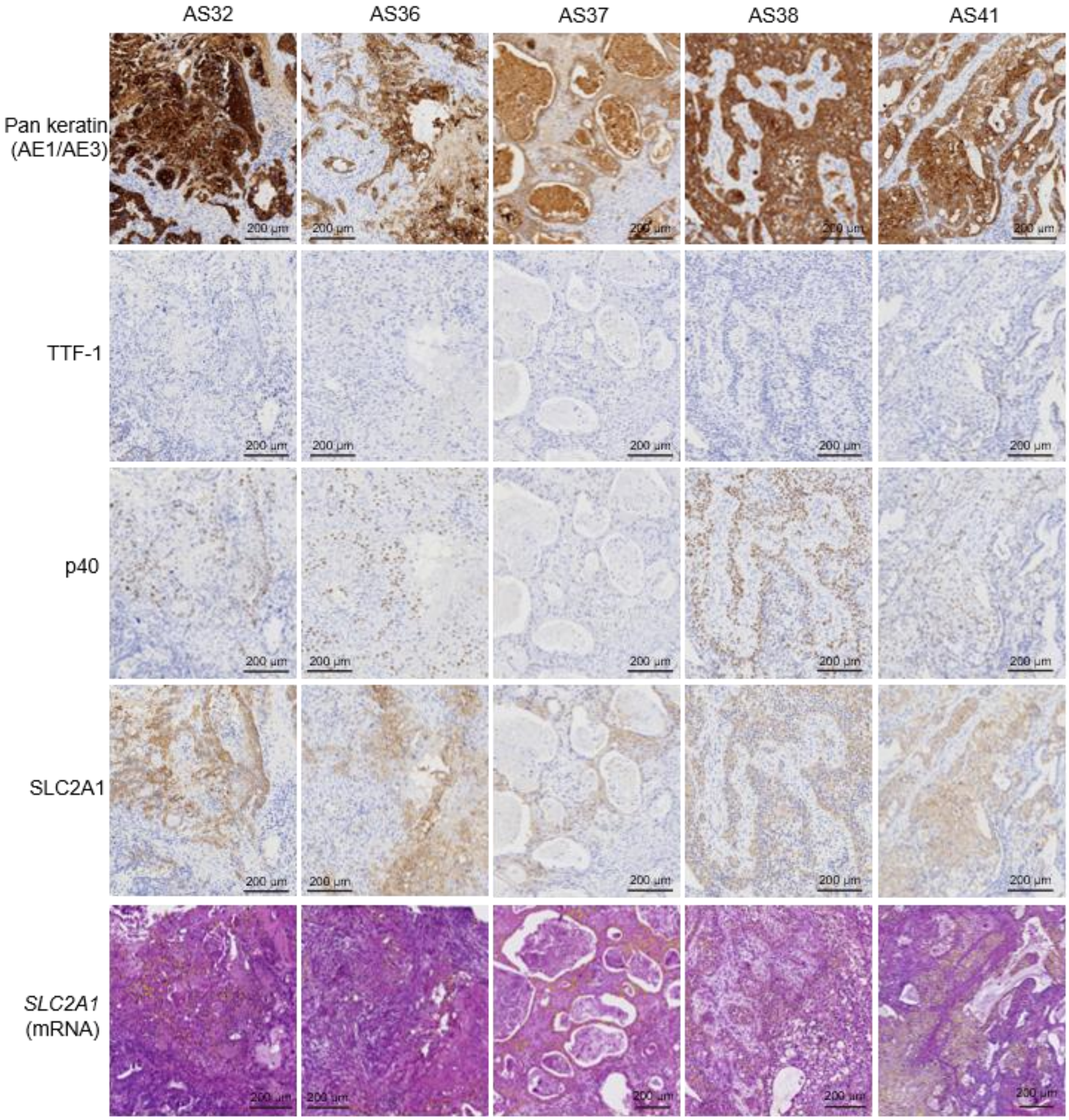


**Supplementary Figure S8. Inter-patient validation of SLC2A1 expression patterns in TTF-1⁻/p40⁻ tumor regions using immunohistochemistry and spatial mRNA mapping.** Serial sections were stained for pan-keratin (AE1/AE3), TTF-1, p40, and SLC2A1 protein, together with spatial maps of *SLC2A1* mRNA expression. Pan-keratin marks epithelial cell populations, whereas TTF-1 and p40 distinguish adenocarcinoma and squamous differentiation, respectively. *SLC2A1* expression signals as yellow dots overlaid on the H&E-stained section. SLC2A1 protein staining and spatial mRNA expression localized to the same tumor regions, including TTF-1⁻/p40⁻ tumor areas and squamous cell regions that were p40 positive. Scale bars = 200 µm.

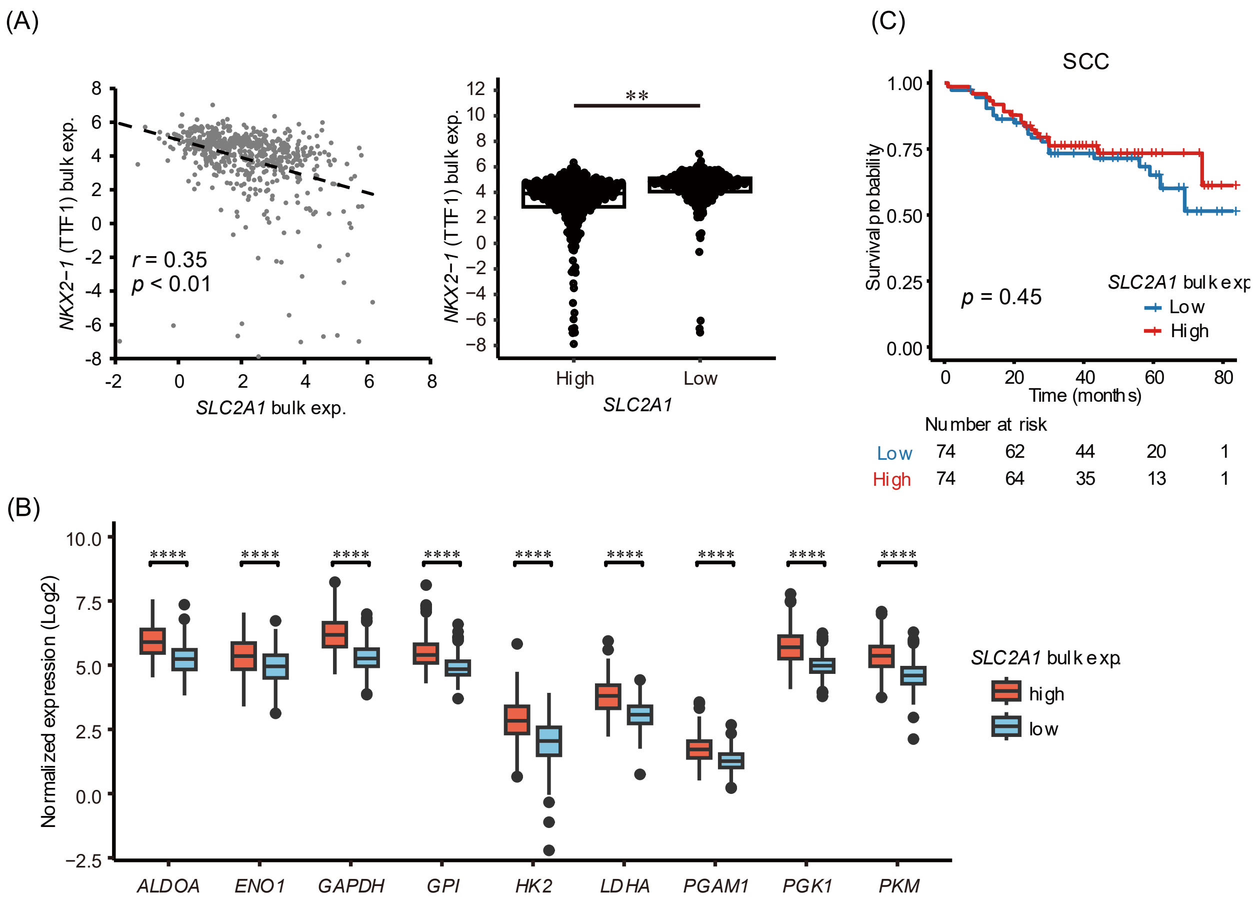


**Supplementary Figure S9. Relationship between *SLC2A1* bulk expression, *NKX2-1* (*TTF-1*) bulk expression, and glycolysis-related gene bulk expression in lung adenocarcinoma (ADC), and survival analysis for squamous cell carcinoma (SCC).** (A) Left: Scatter plot showing the correlation between bulk expression (exp) levels of SLC2A1 and *NKX2-1* in ADC. Right: Comparison of *NKX2-1* bulk expression between the *SLC2A1*-high and *SLC2A1*-low groups. Asterisks indicate statistical significance (**$p$ < 0.01). (B) Comparison of normalized log2 expression levels of glycolysis-related genes (*ALDOA*, *ENO1*, *GAPDH*, *GPI*, *HK2*, *LDHA*, *PGAM1*, *PGK1*, *PKM*) between *SLC2A1*-high and *SLC2A1*-low ADC groups (****$p$ < 0.0001). (C) Kaplan–Meier survival curves of overall survival in patients with SCC stratified by *SLC2A1* bulk expression (high vs. low).

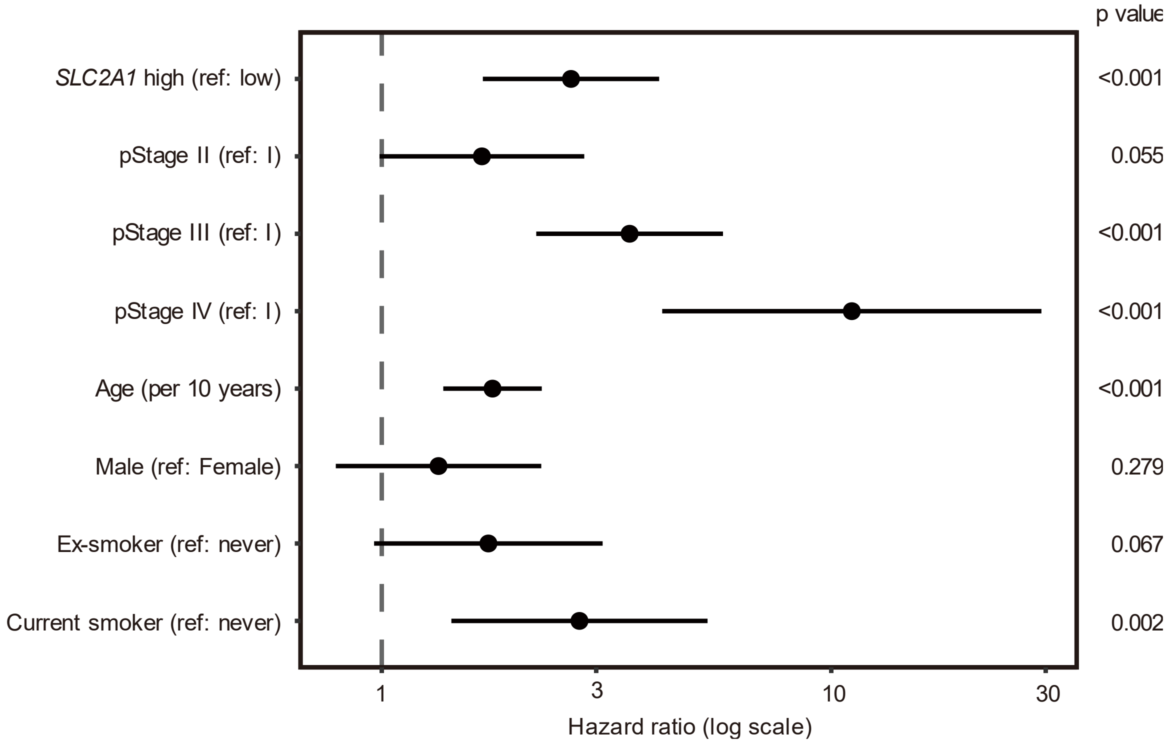


**Supplementary Figure S10. Multivariable Cox proportional hazards analysis of overall survival in lung adenocarcinoma.** pStage, pathological stage; ref, reference.

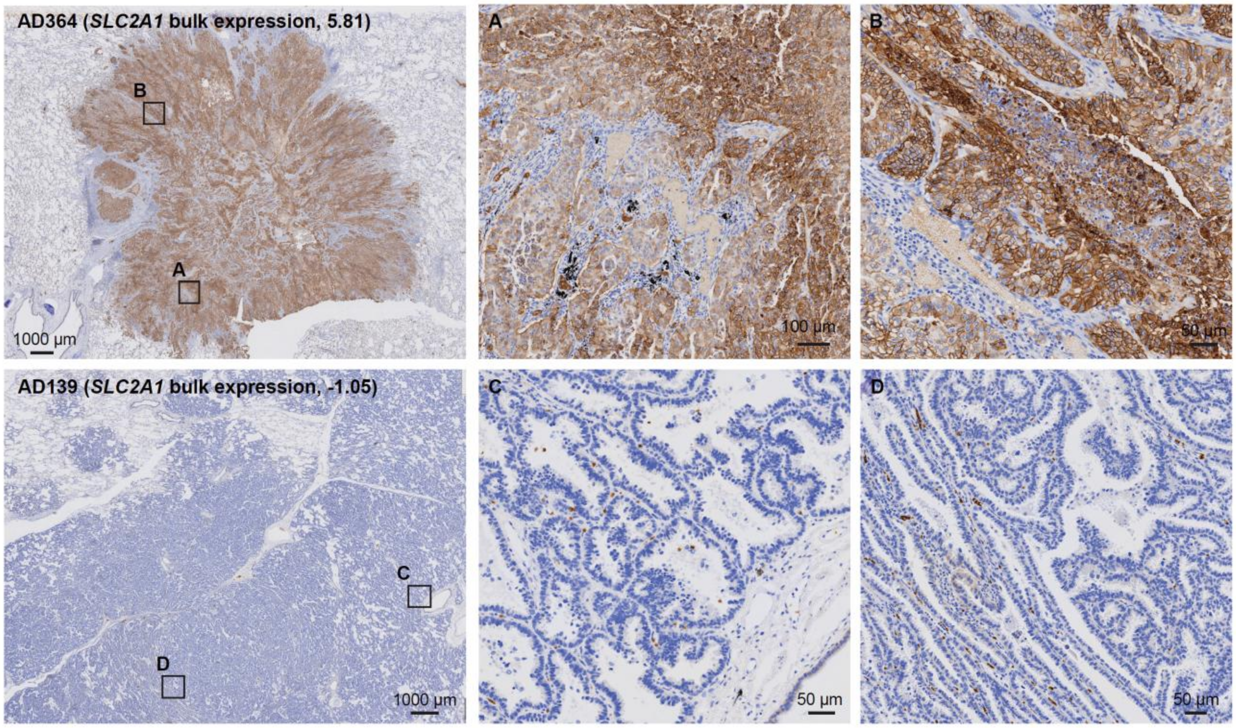


**Supplementary Figure S11. Immunohistochemical validation of SLC2A1 expression in ADC cases.** Representative immunohistochemical images from two independent lung adenocarcinoma (ADC) samples stratified by *SLC2A1* bulk expression. (A) In the *SLC2A1*-high case (AD364), strong membranous and cytoplasmic staining was observed in tumor epithelial regions. (B) In contrast, the *SLC2A1*-low case (AD139) showed only weak or focal staining.

**Supplementary Table S1. Probe information for Xenium spatial transcriptomics**

| Gene | Ensembl ID | Probesets | Gene | Ensembl ID | Probesets |
|---|---|---|---|---|---|
| CDC20 | ENSG00000117399 | 8 | ENAH | ENSG00000154380 | 8 |
| PLK1 | ENSG00000166851 | 8 | EPCAM | ENSG00000119888 | 4 |
| CENPA | ENSG00000115163 | 8 | ETV5 | ENSG00000244405 | 8 |
| KIF2C | ENSG00000142945 | 8 | F3 | ENSG00000117525 | 8 |
| ACTL6A | ENSG00000136518 | 8 | FABP3 | ENSG00000121769 | 8 |
| ELAVL1 | ENSG00000066044 | 8 | FAM184A | ENSG00000111879 | 8 |
| MAD2L1 | ENSG00000164109 | 8 | FAS | ENSG00000026103 | 8 |
| NEK2 | ENSG00000117650 | 8 | FASLG | ENSG00000117560 | 8 |
| OIP5 | ENSG00000104147 | 8 | FASN | ENSG00000169710 | 8 |
| VIM | ENSG00000026025 | 8 | FBN1 | ENSG00000166147 | 8 |
| ANXA6 | ENSG00000197043 | 8 | FCER1A | ENSG00000179639 | 8 |
| LIX1L | ENSG00000271601 | 8 | FCGR1A | ENSG00000150337 | 8 |
| CNRIP1 | ENSG00000119865 | 8 | FCGR3A | ENSG00000203747 | 8 |
| DAB2 | ENSG00000153071 | 8 | FCMR | ENSG00000162894 | 8 |
| COL6A1 | ENSG00000142156 | 8 | FCN1 | ENSG00000085265 | 8 |
| ZEB2 | ENSG00000169554 | 8 | FCN3 | ENSG00000142748 | 6 |
| COL6A2 | ENSG00000142173 | 8 | FGFBP2 | ENSG00000137441 | 8 |
| ST3GAL2 | ENSG00000157350 | 8 | FGFR4 | ENSG00000160867 | 7 |
| CMTM3 | ENSG00000140931 | 8 | FKBP11 | ENSG00000134285 | 5 |
| PMP22 | ENSG00000109099 | 8 | FOXI1 | ENSG00000168269 | 8 |
| LAMA4 | ENSG00000112769 | 8 | FOXJ1 | ENSG00000129654 | 4 |
| CDH2 | ENSG00000170558 | 8 | FOXP3 | ENSG00000049768 | 7 |
| FSTL1 | ENSG00000163430 | 8 | FSCN1 | ENSG00000075618 | 3 |
| COL5A1 | ENSG00000130635 | 8 | GJA5 | ENSG00000265107 | 8 |
| POSTN | ENSG00000133110 | 8 | GKN2 | ENSG00000183607 | 8 |
| COL6A3 | ENSG00000163359 | 8 | GLCCI1 | ENSG00000106415 | 8 |
| SPARC | ENSG00000113140 | 8 | GLIPR2 | ENSG00000122694 | 8 |
| ESRP1 | ENSG00000104413 | 8 | GNG11 | ENSG00000127920 | 8 |
| OVOL1 | ENSG00000172818 | 8 | GPI | ENSG00000105220 | 7 |
| GRHL2 | ENSG00000083307 | 8 | GPR171 | ENSG00000174946 | 8 |
| ERBB3 | ENSG00000065361 | 8 | GPR183 | ENSG00000169508 | 8 |
| ESRP2 | ENSG00000103067 | 8 | GPR34 | ENSG00000171659 | 8 |
| CDS1 | ENSG00000163624 | 8 | GPX2 | ENSG00000176153 | 8 |
| EPN3 | ENSG00000049283 | 8 | GZMA | ENSG00000145649 | 8 |
| EPHA1 | ENSG00000146904 | 8 | GZMB | ENSG00000100453 | 7 |
| GRHL3 | ENSG00000158055 | 8 | GZMK | ENSG00000113088 | 8 |
| GRHL1 | ENSG00000134317 | 8 | HAVCR2 | ENSG00000135077 | 8 |
| PSAT1 | ENSG00000135069 | 8 | HIF1A | ENSG00000100644 | 8 |
| TFRC | ENSG00000072274 | 8 | HIGD1B | ENSG00000131097 | 5 |
| EGFL7 | ENSG00000172889 | 8 | HMGCS1 | ENSG00000112972 | 8 |
| UPF3B | ENSG00000125351 | 8 | HP | ENSG00000257017 | 8 |
| AXL | ENSG00000167601 | 8 | HPGDS | ENSG00000163106 | 8 |
| ZEB1 | ENSG00000148516 | 8 | ICA1 | ENSG00000003147 | 8 |

| | | | | | |
|---|---|---|---|---|---|
| YAP1 | ENSG00000137693 | 8 | IGF1 | ENSG00000017427 | 8 |
| FOXM1 | ENSG00000111206 | 8 | IGFBP3 | ENSG00000146674 | 4 |
| SRSF2 | ENSG00000161547 | 8 | IL1RL1 | ENSG00000115602 | 8 |
| SRSF3 | ENSG00000112081 | 8 | IL7R | ENSG00000168685 | 8 |
| SRSF7 | ENSG00000115875 | 8 | IQGAP2 | ENSG00000145703 | 8 |
| CCNE1 | ENSG00000105173 | 8 | IRF8 | ENSG00000140968 | 8 |
| RB1 | ENSG00000139687 | 8 | ITGAM | ENSG00000169896 | 8 |
| CDKN2A | ENSG00000147889 | 8 | ITGB4 | ENSG00000132470 | 7 |
| CDKN2B | ENSG00000147883 | 8 | KCNK3 | ENSG00000171303 | 8 |
| ERBB2 | ENSG00000141736 | 8 | KDR | ENSG00000128052 | 8 |
| TERT | ENSG00000164362 | 8 | KIT | ENSG00000157404 | 8 |
| GNAS | ENSG00000087460 | 8 | KLF5 | ENSG00000102554 | 4 |
| PTEN | ENSG00000171862 | 8 | KLK11 | ENSG00000167757 | 5 |
| NOTCH1 | ENSG00000148400 | 8 | KLRB1 | ENSG00000111796 | 8 |
| CTNNB1 | ENSG00000168036 | 8 | KLRC1 | ENSG00000134545 | 8 |
| MAP3K1 | ENSG00000095015 | 8 | KLRD1 | ENSG00000134539 | 8 |
| MDM2 | ENSG00000135679 | 8 | KRT15 | ENSG00000171346 | 4 |
| KEAP1 | ENSG00000079999 | 8 | KRT7 | ENSG00000135480 | 4 |
| NF1 | ENSG00000196712 | 8 | LAG3 | ENSG00000089692 | 5 |
| SMARCA4 | ENSG00000127616 | 8 | LAMC3 | ENSG00000050555 | 8 |
| CDH23 | ENSG00000107736 | 8 | LCK | ENSG00000182866 | 8 |
| ARAF | ENSG00000078061 | 8 | LGALS3BP | ENSG00000108679 | 4 |
| BRD3 | ENSG00000169925 | 8 | LGR5 | ENSG00000139292 | 4 |
| NOTCH3 | ENSG00000074181 | 8 | LGR6 | ENSG00000133067 | 8 |
| EZH2 | ENSG00000106462 | 8 | LILRA4 | ENSG00000239961 | 8 |
| TTF1 | ENSG00000125482 | 8 | LILRA5 | ENSG00000187116 | 5 |
| FGFR1 | ENSG00000077782 | 8 | LILRB2 | ENSG00000131042 | 8 |
| CCND1 | ENSG00000110092 | 8 | LILRB4 | ENSG00000186818 | 4 |
| MDM4 | ENSG00000198625 | 8 | LMOD1 | ENSG00000163431 | 8 |
| PPM1D | ENSG00000170836 | 8 | LTBP2 | ENSG00000119681 | 8 |
| CSMD1 | ENSG00000183117 | 8 | LTF | ENSG00000012223 | 4 |
| WWOX | ENSG00000186153 | 8 | LYVE1 | ENSG00000133800 | 8 |
| LRP1B | ENSG00000168702 | 8 | MALL | ENSG00000144063 | 4 |
| FAT2 | ENSG00000086570 | 8 | MAP7 | ENSG00000135525 | 8 |
| TGM1 | ENSG00000092295 | 8 | MARCO | ENSG00000019169 | 8 |
| P2RY1 | ENSG00000169860 | 8 | MCEMP1 | ENSG00000183019 | 8 |
| FST | ENSG00000134363 | 8 | MEDAG | ENSG00000102802 | 8 |
| MUC21 | ENSG00000204544 | 8 | MET | ENSG00000105976 | 8 |
| KPNA7 | ENSG00000185467 | 8 | MFAP5 | ENSG00000197614 | 8 |
| OR7C2 | ENSG00000127529 | 8 | MIS18BP1 | ENSG00000129534 | 8 |
| MMP15 | ENSG00000102996 | 8 | MKI67 | ENSG00000148773 | 8 |
| MACC1 | ENSG00000183742 | 8 | MMP12 | ENSG00000262406 | 4 |
| CLEC4G | ENSG00000182566 | 8 | MMP9 | ENSG00000100985 | 6 |
| VEGFC | ENSG00000150630 | 8 | MMRN1 | ENSG00000138722 | 8 |
| CD209 | ENSG00000090659 | 8 | MPEG1 | ENSG00000197629 | 8 |
| MCM2 | ENSG00000073111 | 8 | MS4A1 | ENSG00000156738 | 8 |

| | | | | | |
|---|---|---|---|---|---|
| MCM6 | ENSG00000076003 | 8 | MS4A2 | ENSG00000149534 | 8 |
| CHAF1A | ENSG00000167670 | 8 | MS4A4A | ENSG00000110079 | 8 |
| RFC4 | ENSG00000163918 | 8 | MTUS1 | ENSG00000129422 | 8 |
| CENPE | ENSG00000138778 | 8 | MUC1 | ENSG00000185499 | 4 |
| BUB1 | ENSG00000169679 | 8 | MUC5B | ENSG00000117983 | 4 |
| BIRC5 | ENSG00000089685 | 8 | MYC | ENSG00000136997 | 8 |
| EMID1 | ENSG00000186998 | 8 | MYH11 | ENSG00000133392 | 4 |
| PPBP | ENSG00000163736 | 8 | MYO6 | ENSG00000196586 | 8 |
| ITGA2B | ENSG00000005961 | 8 | MZB1 | ENSG00000170476 | 6 |
| TGFB1 | ENSG00000105329 | 8 | NCEH1 | ENSG00000144959 | 8 |
| ITGA2 | ENSG00000164171 | 8 | NFKB1 | ENSG00000109320 | 8 |
| ACE | ENSG00000159640 | 8 | NID1 | ENSG00000116962 | 8 |
| ACE2 | ENSG00000130234 | 8 | NKG7 | ENSG00000105374 | 2 |
| ACKR1 | ENSG00000213088 | 8 | NTN4 | ENSG00000074527 | 4 |
| ADAM17 | ENSG00000151694 | 8 | NTRK2 | ENSG00000148053 | 8 |
| ADAM28 | ENSG00000042980 | 8 | OTUD7B | ENSG00000264522 | 8 |
| ADAMTS1 | ENSG00000154734 | 8 | P2RX1 | ENSG00000108405 | 8 |
| ADGRL4 | ENSG00000162618 | 8 | PAMR1 | ENSG00000149090 | 8 |
| AGER | ENSG00000204305 | 4 | PCNA | ENSG00000132646 | 4 |
| AGR3 | ENSG00000173467 | 8 | PCOLCE2 | ENSG00000163710 | 8 |
| AIF1 | ENSG00000204472 | 7 | PDCD1 | ENSG00000188389 | 2 |
| ANPEP | ENSG00000166825 | 8 | PDCD1LG2 | ENSG00000197646 | 8 |
| APOD | ENSG00000189058 | 7 | PDGFRA | ENSG00000134853 | 8 |
| APOLD1 | ENSG00000178878 | 8 | PDGFRB | ENSG00000113721 | 8 |
| AQP9 | ENSG00000103569 | 8 | PDPN | ENSG00000162493 | 8 |
| AREG | ENSG00000109321 | 8 | PEBP4 | ENSG00000134020 | 6 |
| ARL14 | ENSG00000179674 | 8 | PI3 | ENSG00000124102 | 5 |
| ASCL1 | ENSG00000139352 | 8 | PIM1 | ENSG00000137193 | 8 |
| ASCL2 | ENSG00000183734 | 4 | PIM2 | ENSG00000102096 | 8 |
| ASCL3 | ENSG00000176009 | 7 | PLA2G2A | ENSG00000188257 | 6 |
| ATP1B1 | ENSG00000143153 | 4 | PLA2G4F | ENSG00000168907 | 8 |
| BAIAP2L1 | ENSG00000006453 | 8 | PLA2G7 | ENSG00000146070 | 8 |
| BANK1 | ENSG00000153064 | 8 | PLN | ENSG00000198523 | 8 |
| BCAS1 | ENSG00000064787 | 8 | PLVAP | ENSG00000130300 | 8 |
| BMX | ENSG00000102010 | 8 | POU2AF1 | ENSG00000110777 | 8 |
| CA4 | ENSG00000167434 | 5 | PROX1 | ENSG00000117707 | 8 |
| CCDC78 | ENSG00000162004 | 3 | PTGS1 | ENSG00000095303 | 8 |
| CCNA1 | ENSG00000133101 | 8 | RAMP2 | ENSG00000131477 | 4 |
| CCNB2 | ENSG00000157456 | 8 | RARRES1 | ENSG00000118849 | 4 |
| CCR7 | ENSG00000126353 | 8 | RBP4 | ENSG00000138207 | 8 |
| CD14 | ENSG00000170458 | 8 | RERGL | ENSG00000111404 | 8 |
| CD163 | ENSG00000177575 | 4 | RETN | ENSG00000104918 | 1 |
| CD19 | ENSG00000177455 | 8 | RGS5 | ENSG00000143248 | 4 |
| CD1A | ENSG00000158477 | 8 | RND1 | ENSG00000172602 | 8 |
| CD1C | ENSG00000158481 | 8 | RUNX3 | ENSG00000020633 | 8 |
| CD2 | ENSG00000116824 | 8 | S100A12 | ENSG00000163221 | 5 |

| | | | | | |
|---|---|---|---|---|---|
| CD24 | ENSG00000272398 | 3 | S100A7 | ENSG00000143556 | 4 |
| CD247 | ENSG00000198821 | 8 | S100B | ENSG00000160307 | 8 |
| CD27 | ENSG00000139193 | 5 | SAMD3 | ENSG00000164483 | 8 |
| CD274 | ENSG00000120217 | 8 | SCEL | ENSG00000136155 | 8 |
| CD28 | ENSG00000178562 | 8 | SEC11C | ENSG00000166562 | 8 |
| CD300E | ENSG00000186407 | 8 | SELE | ENSG00000007908 | 8 |
| CD34 | ENSG00000174059 | 8 | SELL | ENSG00000188404 | 8 |
| CD38 | ENSG00000004468 | 8 | SELP | ENSG00000174175 | 8 |
| CD3D | ENSG00000167286 | 5 | SEMA3B | ENSG00000012171 | 4 |
| CD3E | ENSG00000198851 | 8 | SEMA3C | ENSG00000075223 | 8 |
| CD4 | ENSG00000010610 | 8 | SERPINA3 | ENSG00000196136 | 8 |
| CD40 | ENSG00000101017 | 7 | SFRP2 | ENSG00000145423 | 4 |
| CD40LG | ENSG00000102245 | 8 | SFTA2 | ENSG00000196260 | 6 |
| CD68 | ENSG00000129226 | 4 | SFTPD | ENSG00000133661 | 8 |
| CD70 | ENSG00000125726 | 2 | SHANK3 | ENSG00000251322 | 8 |
| CD79A | ENSG00000105369 | 4 | SLC15A2 | ENSG00000163406 | 8 |
| CD80 | ENSG00000121594 | 8 | SLC18A2 | ENSG00000165646 | 8 |
| CD86 | ENSG00000114013 | 8 | SLC1A3 | ENSG00000079215 | 8 |
| CD8A | ENSG00000153563 | 8 | SLC2A1 | ENSG00000117394 | 4 |
| CD8B | ENSG00000172116 | 8 | SLC7A11 | ENSG00000151012 | 8 |
| CDH1 | ENSG00000039068 | 8 | SLIT3 | ENSG00000184347 | 8 |
| CDK1 | ENSG00000170312 | 8 | SMIM24 | ENSG00000095932 | 8 |
| CENPF | ENSG00000117724 | 8 | SOX2 | ENSG00000181449 | 8 |
| CFB | ENSG00000243649 | 8 | SOX9 | ENSG00000125398 | 8 |
| CFTR | ENSG00000001626 | 8 | SPDEF | ENSG00000124664 | 6 |
| CHIT1 | ENSG00000133063 | 4 | SPIB | ENSG00000269404 | 8 |
| CLDN5 | ENSG00000184113 | 6 | STAT4 | ENSG00000138378 | 8 |
| CLEC10A | ENSG00000132514 | 8 | STC1 | ENSG00000159167 | 8 |
| CLEC12A | ENSG00000172322 | 8 | STEAP4 | ENSG00000127954 | 8 |
| CLEC4E | ENSG00000166523 | 8 | SVEP1 | ENSG00000165124 | 8 |
| CNN1 | ENSG00000130176 | 6 | SYK | ENSG00000165025 | 8 |
| COL5A2 | ENSG00000204262 | 8 | TACSTD2 | ENSG00000184292 | 4 |
| COL8A1 | ENSG00000144810 | 8 | TC2N | ENSG00000165929 | 8 |
| CP | ENSG00000047457 | 4 | TCL1A | ENSG00000100721 | 7 |
| CSPG4 | ENSG00000173546 | 8 | THBS2 | ENSG00000186340 | 8 |
| CSTA | ENSG00000121552 | 4 | THY1 | ENSG00000154096 | 8 |
| CTLA4 | ENSG00000163599 | 8 | TM4SF18 | ENSG00000163762 | 8 |
| CTSL | ENSG00000135047 | 4 | TM4SF4 | ENSG00000169903 | 8 |
| CTTN | ENSG00000085733 | 8 | TMC5 | ENSG00000103534 | 8 |
| CXCL10 | ENSG00000169245 | 8 | TMEM100 | ENSG00000166292 | 8 |
| CXCL13 | ENSG00000156234 | 8 | TMPRSS2 | ENSG00000184012 | 8 |
| CXCL5 | ENSG00000163735 | 8 | TNFRSF13B | ENSG00000240505 | 5 |
| CXCL6 | ENSG00000124875 | 8 | TNFRSF13C | ENSG00000159958 | 8 |
| CXCL9 | ENSG00000138755 | 8 | TNFRSF17 | ENSG00000048462 | 8 |
| CXCR5 | ENSG00000160683 | 8 | TNFRSF18 | ENSG00000186891 | 1 |
| CXCR6 | ENSG00000172215 | 8 | TOP2A | ENSG00000131747 | 8 |

| | | | | | |
|---|---|---|---|---|---|
| CYP2F1 | ENSG00000197446 | 8 | TP63 | ENSG00000073282 | 4 |
| DAPK2 | ENSG00000035664 | 8 | TP73 | ENSG00000078900 | 8 |
| DCLK1 | ENSG00000133083 | 8 | TREM2 | ENSG00000095970 | 7 |
| DES | ENSG00000175084 | 8 | TRPC6 | ENSG00000137672 | 8 |
| DGKG | ENSG00000058866 | 8 | TSPAN8 | ENSG00000127324 | 4 |
| DIRAS3 | ENSG00000162595 | 8 | UBE2C | ENSG00000175063 | 4 |
| DMBT1 | ENSG00000187908 | 8 | UPK1B | ENSG00000114638 | 8 |
| DNAJB9 | ENSG00000128590 | 8 | UPK3B | ENSG00000243566 | 4 |
| DPP6 | ENSG00000130226 | 8 | VSIG4 | ENSG00000155659 | 8 |
| DUOX1 | ENSG00000137857 | 8 | VWF | ENSG00000110799 | 8 |
| ECSCR | ENSG00000249751 | 8 | WFS1 | ENSG00000109501 | 8 |
| EGFR | ENSG00000146648 | 4 | WNT2 | ENSG00000105989 | 8 |
| EHF | ENSG00000135373 | 8 | WT1 | ENSG00000184937 | 8 |
| ELF3 | ENSG00000163435 | 8 | | | |

**Supplementary Table S2. Characteristics of patients with adenocarcinoma (ADC) and squamous cell carcinoma (SCC) along with their survival outcomes**

| Characteristic | ADC (n = 574) | SCC (n = 148) |
|---|---|---|
| Age at diagnosis (y), median ± SD | 70 ± 8.8 | 72 ± 6.9 |
| Sex | | |
| Female | 274 | 18 |
| Male | 300 | 130 |
| Stage | | |
| I | 371 | 75 |
| II | 98 | 50 |
| III | 97 | 22 |
| IV | 8 | 1 |
| Smoking | | |
| Current | 109 | 63 |
| ex- | 227 | 85 |
| never | 238 | 0 |

**SD, standard deviation**

**Supplementary Table S3. Positive cell counts of the 39 tumor-specific genes across adenocarcinoma (ADC), TTF-1⁻/p40⁻, and squamous cell carcinoma (SCC) subgroups in the discovery (AS23) and validation (AS26) samples.**

| Gene | Number of positive cells in each subgroup | | | | | |
|---|---|---|---|---|---|---|
| | AS23 (discovery) | | | AS26 (validation) | | |
| | ADC | TTF-1$^{-}$/p40$^{-}$ | SCC | ADC | TTF-1$^{-}$/p40$^{-}$ | SCC |
| *ADAM17* | 10492 | 3498 | 5666 | 6094 | 2504 | 3921 |
| *ARAF* | 15617 | 710 | 1010 | 7226 | 1446 | 2562 |
| *ATP1B1* | 50153 | 8991 | 8766 | 36630 | 14140 | 14559 |
| *BAIAP2L1* | 19427 | 3159 | 3633 | 6820 | 2002 | 2702 |
| *CCND1* | 27112 | 5909 | 7026 | 6290 | 2320 | 4655 |
| *CDH1* | 82536 | 7418 | 8684 | 25850 | 4773 | 8684 |
| *CFB* | 12476 | 3964 | 997 | 25211 | 702 | 3086 |
| *CP* | 60076 | 6603 | 8034 | 36945 | 3093 | 4973 |
| *CTNNB1* | 27920 | 1474 | 2014 | 6769 | 1572 | 2978 |
| *CTTN* | 46379 | 8209 | 8930 | 17063 | 4802 | 6726 |
| *EHF* | 16693 | 4802 | 1333 | 20242 | 1397 | 5153 |
| *ELAVL1* | 19740 | 3218 | 4447 | 8613 | 1273 | 4406 |
| *ELF3* | 76171 | 9395 | 4320 | 26077 | 1005 | 1788 |
| *EPCAM* | 83567 | 5378 | 3266 | 42133 | 3231 | 13089 |
| *ERBB2* | 39519 | 2073 | 2179 | 13559 | 404 | 1460 |
| *ERBB3* | 43385 | 3138 | 2877 | 13061 | 611 | 1742 |
| *FASN* | 17626 | 2228 | 2603 | 21062 | 1248 | 3055 |
| *ITGA2* | 32808 | 3520 | 2325 | 20440 | 1122 | 3792 |
| *ITGB4* | 18094 | 3302 | 3163 | 23720 | 1178 | 4482 |
| *KRT7* | 204959 | 25963 | 13307 | 74268 | 7239 | 22677 |
| *LGALS3BP* | 22947 | 4447 | 3061 | 18524 | 1886 | 4363 |
| *MACC1* | 35023 | 7670 | 4635 | 18051 | 1570 | 3457 |
| *MALL* | 19448 | 5383 | 1507 | 8188 | 1261 | 1533 |
| *MDM2* | 44607 | 4216 | 2681 | 23698 | 697 | 12437 |
| *MET* | 65717 | 1492 | 3119 | 23465 | 470 | 11663 |
| *MUC1* | 52065 | 3916 | 5772 | 26305 | 1195 | 5772 |
| *MYO6* | 22063 | 2680 | 2131 | 13933 | 1118 | 4913 |
| *NFKB1* | 21393 | 1579 | 2028 | 14903 | 556 | 2681 |
| *OTUD7B* | 21777 | 1450 | 1615 | 18042 | 523 | 1450 |
| *SEMA3C* | 10081 | 2683 | 1478 | 6978 | 658 | 2683 |

| | | | | | | |
|---|---|---|---|---|---|---|
| *SLC2A1* | 20982 | 2675 | 7416 | 10129 | 1610 | 7416 |
| *SMARCA4* | 45686 | 1669 | 3109 | 17726 | 643 | 1669 |
| *SOX9* | 27364 | 2837 | 3566 | 10170 | 1294 | 2837 |
| *SRSF3* | 42051 | 1951 | 2180 | 18637 | 726 | 1951 |
| *SRSF7* | 33588 | 4076 | 6145 | 25497 | 1368 | 4076 |
| *TACSTD2* | 44658 | 8208 | 7561 | 20217 | 2094 | 8208 |
| *TC2N* | 21153 | 3187 | 1309 | 15044 | 1028 | 3187 |
| *TFRC* | 38160 | 4635 | 2976 | 13967 | 1184 | 4635 |
| *YAP1* | 28242 | 4363 | 4431 | 7594 | 1526 | 4363 |

**Supplementary Table S4. Gene Ontology (GO) Biological Process enrichment analysis in SLC2A1-related molecules.**

| Term name | Term ID | Adjusted p value |
|---|---|---|
| **negative regulation of programmed cell death** | **GO:0043069** | **4.02E-09** |
| negative regulation of apoptotic process | GO:0043066 | 6.58E-08 |
| **neurogenesis** | **GO:0022008** | **1.2E-06** |
| animal organ development | GO:0048513 | 1.25E-06 |
| gland development | GO:0048732 | 1.44E-06 |
| regulation of programmed cell death | GO:0043067 | 2.29E-06 |
| **cell adhesion** | **GO:0007155** | **2.8E-06** |
| cell differentiation | GO:0030154 | 3.08E-06 |
| cellular developmental process | GO:0048869 | 3.09E-06 |
| tissue development | GO:0009888 | 7.15E-06 |
| nervous system development | GO:0007399 | 1.62E-05 |
| tissue morphogenesis | GO:0048729 | 2.01E-05 |
| regulation of apoptotic process | GO:0042981 | 2.35E-05 |
| anatomical structure morphogenesis | GO:0009653 | 2.86E-05 |
| **response to xenobiotic stimulus** | **GO:0009410** | **2.9E-05** |
| epithelium development | GO:0060429 | 5.4E-05 |
| anatomical structure development | GO:0048856 | 5.83E-05 |
| programmed cell death | GO:0012501 | 6.63E-05 |
| **tissue remodeling** | **GO:0048771** | **6.7E-05** |
| cell death | GO:0008219 | 6.79E-05 |
| morphogenesis of a branching epithelium | GO:0061138 | 8.96E-05 |
| system development | GO:0048731 | 9.11E-05 |
| **glandular epithelial cell differentiation** | **GO:0002067** | **0.00013** |
| animal organ morphogenesis | GO:0009887 | 0.000139 |
| positive regulation of epithelial cell proliferation | GO:0050679 | 0.000141 |
| morphogenesis of a branching structure | GO:0001763 | 0.000149 |
| positive regulation of macromolecule biosynthetic process | GO:0010557 | 0.000166 |
| regulation of epithelial cell proliferation | GO:0050678 | 0.000185 |
| positive regulation of biosynthetic process | GO:0009891 | 0.000273 |
| neuron differentiation | GO:0030182 | 0.000274 |
| developmental process | GO:0032502 | 0.000299 |
| heart development | GO:0007507 | 0.000343 |
| positive regulation of macromolecule metabolic process | GO:0010604 | 0.000394 |
| negative regulation of epithelial cell differentiation | GO:0030857 | 0.000418 |
| positive regulation of cellular process | GO:0048522 | 0.000481 |
| generation of neurons | GO:0048699 | 0.000489 |
| apoptotic process | GO:0006915 | 0.000491 |
| cell development | GO:0048468 | 0.000617 |
| glial cell differentiation | GO:0010001 | 0.000644 |
| negative regulation of developmental process | GO:0051093 | 0.000668 |
| epithelial cell proliferation | GO:0050673 | 0.000676 |

| | | |
|---|---|---|
| **tissue homeostasis** | **GO:0001894** | **0.0007** |
| anatomical structure homeostasis | GO:0060249 | 0.000719 |
| regulation of developmental process | GO:0050793 | 0.00082 |
| positive regulation of cell population proliferation | GO:0008284 | 0.000866 |
| positive regulation of nucleobase-containing compound metabolic process | GO:0045935 | 0.000916 |
| multicellular organism development | GO:0007275 | 0.000962 |
| positive regulation of biological process | GO:0048518 | 0.001267 |
| **cell growth** | **GO:0016049** | **0.00128** |
| branching morphogenesis of an epithelial tube | GO:0048754 | 0.00129 |
| positive regulation of metabolic process | GO:0009893 | 0.001315 |
| positive regulation of DNA-templated transcription | GO:0045893 | 0.001332 |
| positive regulation of RNA biosynthetic process | GO:1902680 | 0.001348 |
| positive regulation of developmental process | GO:0051094 | 0.001465 |
| morphogenesis of an epithelium | GO:0002009 | 0.001634 |
| epithelial cell differentiation | GO:0030855 | 0.001678 |
| mesenchyme development | GO:0060485 | 0.001979 |
| cellular response to mechanical stimulus | GO:0071260 | 0.002225 |
| skin development | GO:0043588 | 0.002244 |
| cellular response to external stimulus | GO:0071496 | 0.002348 |
| signaling | GO:0023052 | 0.002472 |
| cellular response to stimulus | GO:0051716 | 0.002575 |
| cell communication | GO:0007154 | 0.002637 |
| cellular response to environmental stimulus | GO:0104004 | 0.002766 |
| cellular response to abiotic stimulus | GO:0071214 | 0.002766 |
| positive regulation of RNA metabolic process | GO:0051254 | 0.002901 |
| gliogenesis | GO:0042063 | 0.003439 |
| **ERBB2 signaling pathway** | **GO:0038128** | **0.00356** |
| response to abiotic stimulus | GO:0009628 | 0.003935 |
| response to stress | GO:0006950 | 0.004505 |
| **cell migration** | **GO:0016477** | **0.00492** |
| signal transduction | GO:0007165 | 0.004984 |
| regulation of DNA-templated transcription | GO:0006355 | 0.005101 |
| regulation of RNA biosynthetic process | GO:2001141 | 0.00546 |
| **regulation of cellular component organization** | **GO:0051128** | **0.0062** |
| regulation of cell differentiation | GO:0045595 | 0.006281 |
| **plasma membrane bounded cell projection organization** | **GO:0120036** | **0.00673** |
| regulation of multicellular organismal process | GO:0051239 | 0.006945 |
| epidermis development | GO:0008544 | 0.00698 |
| response to mechanical stimulus | GO:0009612 | 0.007073 |
| intracellular signal transduction | GO:0035556 | 0.00719 |
| lung cell differentiation | GO:0060479 | 0.007316 |
| lung epithelial cell differentiation | GO:0060487 | 0.007316 |
| nephron tubule development | GO:0072080 | 0.008043 |
| epithelial tube branching involved in lung morphogenesis | GO:0060441 | 0.008123 |
| DNA-templated transcription | GO:0006351 | 0.00834 |

| | | |
|---|---|---|
| positive regulation of transcription by RNA polymerase II | GO:0045944 | 0.008483 |
| cell projection organization | GO:0030030 | 0.008552 |
| negative regulation of apoptotic signaling pathway | GO:2001234 | 0.008767 |
| renal tubule development | GO:0061326 | 0.009374 |
| developmental growth involved in morphogenesis | GO:0060560 | 0.010338 |
| response to oxygen-containing compound | GO:1901700 | 0.010512 |
| negative regulation of cell differentiation | GO:0045596 | 0.010999 |
| negative regulation of response to stimulus | GO:0048585 | 0.011148 |
| stem cell differentiation | GO:0048863 | 0.011428 |
| regulation of cell population proliferation | GO:0042127 | 0.011449 |
| response to stimulus | GO:0050896 | 0.011821 |
| cell-cell adhesion | GO:0098609 | 0.012379 |
| columnar/cuboidal epithelial cell differentiation | GO:0002065 | 0.012956 |
| negative regulation of signal transduction | GO:0009968 | 0.013204 |
| regulation of RNA metabolic process | GO:0051252 | 0.013248 |
| regulation of canonical Wnt signaling pathway | GO:0060828 | 0.013875 |
| cell morphogenesis | GO:0000902 | 0.014366 |
| negative regulation of cellular component organization | GO:0051129 | 0.014638 |
| Wnt signaling pathway | GO:0016055 | 0.015579 |
| **regulation of cellular component biogenesis** | **GO:0044087** | **0.01603** |
| heart morphogenesis | GO:0003007 | 0.017023 |
| nephron epithelium development | GO:0072009 | 0.017461 |
| multicellular organismal process | GO:0032501 | 0.017515 |
| **regulation of protein catabolic process at postsynapse, modulating synaptic transmission** | **GO:0099576** | **0.01947** |
| **cellular response to indole-3-methanol** | **GO:0071681** | **0.01947** |
| cell motility | GO:0048870 | 0.0199 |
| ERBB signaling pathway | GO:0038127 | 0.020413 |
| gland morphogenesis | GO:0022612 | 0.021045 |
| glial cell development | GO:0021782 | 0.023718 |
| digestive tract development | GO:0048565 | 0.024424 |
| Schwann cell development | GO:0014044 | 0.024452 |
| cellular response to epidermal growth factor stimulus | GO:0071364 | 0.024452 |
| negative regulation of signaling | GO:0023057 | 0.025026 |
| skin epidermis development | GO:0098773 | 0.025145 |
| negative regulation of cell communication | GO:0010648 | 0.025167 |
| positive regulation of protein localization | GO:1903829 | 0.025346 |
| response to chemical | GO:0042221 | 0.026749 |
| negative regulation of cell adhesion | GO:0007162 | 0.02714 |
| mammary gland development | GO:0030879 | 0.027402 |
| lung epithelium development | GO:0060428 | 0.028073 |
| **positive regulation of G1/S transition of mitotic cell cycle** | **GO:1900087** | **0.02807** |
| cell surface receptor signaling pathway | GO:0007166 | 0.02902 |
| regulation of branching involved in lung morphogenesis | GO:0061046 | 0.029184 |
| response to indole-3-methanol | GO:0071680 | 0.029184 |
| tube development | GO:0035295 | 0.029837 |

| | | |
|---|---|---|
| regulation of cell adhesion | GO:0030155 | 0.032846 |
| digestive system development | GO:0055123 | 0.033245 |
| response to endogenous stimulus | GO:0009719 | 0.033672 |
| response to epidermal growth factor | GO:0070849 | 0.034141 |
| liver development | GO:0001889 | 0.034149 |
| regulation of nucleobase-containing compound metabolic process | GO:0019219 | 0.034158 |
| canonical Wnt signaling pathway | GO:0060070 | 0.036143 |
| exocrine system development | GO:0035272 | 0.03634 |
| semaphorin-plexin signaling pathway | GO:0071526 | 0.03634 |
| hepaticobiliary system development | GO:0061008 | 0.036969 |
| Schwann cell differentiation | GO:0014037 | 0.038631 |
| circulatory system development | GO:0072359 | 0.039618 |
| **protein localization to nucleus** | **GO:0034504** | **0.04021** |
| response to heparin | GO:0071503 | 0.040829 |
| kidney epithelium development | GO:0072073 | 0.040991 |
| **DNA damage response, signal transduction by p53 class mediator** | **GO:0030330** | **0.04101** |
| regulation of signaling | GO:0023051 | 0.043104 |
| regulation of epithelial cell differentiation | GO:0030856 | 0.043119 |
| endocardial cushion development | GO:0003197 | 0.043493 |
| regulation of cell communication | GO:0010646 | 0.044212 |
| myelination | GO:0042552 | 0.044212 |
| regulation of morphogenesis of a branching structure | GO:0060688 | 0.046068 |
| ensheathment of neurons | GO:0007272 | 0.04646 |
| axon ensheathment | GO:0008366 | 0.04646 |
| nephron development | GO:0072006 | 0.047615 |
| regulation of Wnt signaling pathway | GO:0030111 | 0.048711 |
| cellular response to oxygen-containing compound | GO:1901701 | 0.049821 |

Significantly enriched GO:BP terms identified by g:Profiler are listed, including the term name, GO identifier, and adjusted p value. Driver terms are shown in bold to indicate representative biological processes selected to reduce redundancy within the GO hierarchy.
